# Risks of digestive diseases in long COVID: evidence from a population-based cohort study

**DOI:** 10.1186/s12916-023-03236-4

**Published:** 2024-01-10

**Authors:** Yuying Ma, Lijun Zhang, Rui Wei, Weiyu Dai, Ruijie Zeng, Dongling Luo, Rui Jiang, Zewei Zhuo, Qi Yang, Jingwei Li, Felix W Leung, Chongyang Duan, Weihong Sha, Hao Chen

**Affiliations:** 1 Department of Gastroenterology, Guangdong Provincial People’s Hospital (Guangdong Academy of Medical Sciences), Southern Medical University, Guangzhou, 510080 China; 2 The Second School of Clinical Medicine, Southern Medical University, Guangzhou, 510515 China; 3 School of Medicine, South China University of Technology, Guangzhou, 510006 China; 4 Shantou University Medical College, Shantou, 515000 Guangdong China; 5 Guangdong Provincial People’s Hospital (Guangdong Academy of Medical Sciences), Guangdong Cardiovascular Institute, Southern Medical University, Guangzhou, 510080 China; 6 David Geffen School of Medicine, University of California Los Angeles, Los Angeles, CA 90024 USA; 7 Sepulveda Ambulatory Care Center, Veterans Affairs Greater Los Angeles Healthcare System, North Hills, CA 91343 USA; 8 Department of Biostatistics, School of Public Health, Southern Medical University, Guangzhou, 510515 China

**Keywords:** Gastroenterology, Long-COVID, SARS-CoV-2

## Abstract

**Background:**

In the post-pandemic era, a wide range of COVID-19 sequelae is of growing health concern. However, the risks of digestive diseases in long COVID have not been comprehensively understood. To investigate the long-term risk of digestive diseases among COVID patients.

**Methods:**

In this large-scale retrospective cohort study with up to 2.6 years follow-up (median follow-up: 0.7 years), the COVID-19 group (*n* = 112,311), the contemporary comparison group (*n* = 359,671) and the historical comparison group (*n* = 370,979) predated the COVID-19 outbreak were built using UK Biobank database. Each digestive outcome was defined as the diagnosis 30 days or more after the onset of COVID-19 infection or the index date. Hazard ratios (HRs) and corresponding 95% confidence intervals (CI) were computed utilizing the Cox regression models after inverse probability weighting.

**Results:**

Compared with the contemporary comparison group, patients with previous COVID-19 infection had higher risks of digestive diseases, including gastrointestinal (GI) dysfunction (HR 1.38 (95% CI 1.26 to 1.51)); peptic ulcer disease (HR 1.23 (1.00 to 1.52)); gastroesophageal reflux disease (GERD) (HR 1.41 (1.30 to 1.53)); gallbladder disease (HR 1.21 (1.06 to 1.38)); severe liver disease (HR 1.35 (1.03 to 1.76)); non-alcoholic liver disease (HR 1.27 (1.09 to 1.47)); and pancreatic disease (HR 1.36 (1.11 to 1.66)). The risks of GERD were increased stepwise with the severity of the acute phase of COVID-19 infection. Even after 1-year follow-up, GERD (HR 1.64 (1.30 to 2.07)) and GI dysfunction (HR 1.35 (1.04 to 1.75)) continued to pose risks to COVID-19 patients. Compared to those with one SARS-CoV-2 infection, reinfected patients were at a higher risk of pancreatic diseases (HR 2.57 (1.23 to 5.38)). The results were consistent when the historical cohort was used as the comparison group.

**Conclusions:**

Our study provides insights into the association between COVID-19 and the long-term risk of digestive system disorders. COVID-19 patients are at a higher risk of developing digestive diseases. The risks exhibited a stepwise escalation with the severity of COVID-19, were noted in cases of reinfection, and persisted even after 1-year follow-up. This highlights the need to understand the varying risks of digestive outcomes in COVID-19 patients over time, particularly those who experienced reinfection, and develop appropriate follow-up strategies.

**Supplementary Information:**

The online version contains supplementary material available at 10.1186/s12916-023-03236-4.

## Background

The ongoing pandemic caused by the severe acute respiratory syndrome coronavirus 2 (SARS-CoV-2), commonly referred to as coronavirus disease 2019 (COVID-19), has become a serious global public health concern [1]. As we move into the post-pandemic era, there is growing global attention towards the enduring consequences of COVID-19.

During the recovery period, many COVID-19 patients suffer prolonged multisystem symptoms [2]. According to the US Centers for Disease Control and Prevention, long COVID is a wide range of ongoing health problems that people who have been infected can experience long-term effects from their initial infection [3]. COVID-19 patients who survived the acute phase are at an increased risk of developing cardiovascular diseases [4], kidney dysfunctions [5], and metabolic diseases in the long term [6, 7]. Therefore, it is crucial to pay more attention to post-acute COVID-19 syndrome and its associated complications.

Accumulating evidence suggested that COVID-19 patients experience gastrointestinal symptoms during the acute phase, with the prevalence of all digestive symptoms reported to be up to 9.8% (10.4% for diarrhea, 7.7% for nausea and 6.9% for abdominal pain, respectively) [8–10]. However, the risks of digestive diseases in the post-acute phase of COVID-19 remain unclear. Several studies on the risks of digestive diseases in long COVID have been limited to hospitalized patients with small sample sizes and short-term follow-up [2, 11–13]. Only one large-scale study has extended the analysis to report the 1-year risks and burdens of digestive outcomes in COVID-19 patients [14]. Nevertheless, the generalizability of the findings was limited and compromised by the sex-selective bias resulting from the recruitment of participants from the male-dominant (nearly 90%) US Department of Veterans Affairs database. To overcome the previously mentioned shortcomings, we utilized the UK Biobank with the main strength of a relatively balanced representation of both male and female participants. Furthermore, although sex differences have been reported in COVID-19-related outcomes, the impact of sex on the occurrence of digestive diseases in long COVID remains unknown [15, 16]. Additionally, vaccination against COVID-19 may have an impact on the risk of complications from the disease [17–19]. This issue has not been well resolved in previous studies. Moreover, further investigation is urgently needed to ascertain whether reinfection with SARS-CoV-2 contributes to a pronounced risk of digestive diseases.

In this study, we designed a large-scale population-based retrospective cohort study with long follow-up based on UK Biobank to investigate the long-term hazard of digestive diseases among COVID patients. We also estimated the risk across different follow-up durations, COVID-19 reinfection, and the severity of COVID-19 infection.

## Methods

### Study population and design

Five hundred two thousand, three hundred sixty-eight individuals aged 37 to 73 were enrolled from the general population between 2006 and 2010 throughout the UK in the UK Biobank database. The UK biobank database provided comprehensive health-related information through baseline or follow-up online questionnaires, verbal interviews, biological samples, and physical measurements. Hospital inpatient data were updated regularly by linking to Hospital Episode Statistics (HES) for England, Scottish Morbidity Record for Scotland, and Patient Episode Database for Wales. Death data were acquired through linkage to National Health Service (NHS) Digital and NHS Central Register. COVID-19 testing results records were available (using RT-PCR testing on nasopharyngeal swab specimens) by linking with Public Health England (PHE), Public Health Scotland, and Secure Anonymous Information Linkage in the UK Biobank database with the purpose of conducting COVID-19-related research. In addition, the UK Biobank obtained updated primary care data for roughly 450,000 individuals from two major General Practice (GP) data system providers, EMIS and TPP in England. Further details about the UK Biobank can be found elsewhere [20].

### Definition of COVID-19 infection and comparison groups

We defined COVID-19 infection as the first positive result on COVID-19 polymerase chain reaction (PCR) testing or patients firstly diagnosed with COVID-19 (U07.1 and U07.2) in medical records between 30 January 2020 and 30 October 2022. PHE, Public Health Scotland, and Secure Anonymous Information Linkage provide COVID-19 testing results from Pillar 1 (swab testing of patients with clinical need or serving as healthcare professionals in PHE laboratories and NHS hospitals) and Pillar 2 (swab testing of the wider population). As using hospitalization records alone as a proxy for the severity of infection is not a very reliable or nuanced measure, severe COVID-19 cases were defined as patients who had a critical care admission within 7 days of COVID-19 diagnosis and/or receipt of invasive or non-invasive mechanical ventilation or other respiratory support treatments (including continuous positive airway pressure and oxygen therapy), based on previous studies [4]. OPCS Classification of Interventions and Procedures version 4 system was employed for the identification of these treatments (Additional file 1: Table S1).

To investigate the impact of COVID-19, two comparison cohorts — historical and contemporary comparisons — were included. People who lived during the same period of recruitment as those in the COVID-19 group were included in the contemporary comparisons. The historical comparisons were constructed after excluding individuals in the COVID-19 group and started from January 31^st^, 2017, and ended on October 30^th^, 2019 (3 years before COVID-19). Individuals who died (*n* = 28,980) or were lost to follow-up (*n* = 1298) before January 30^th^, 2020 (the outbreak of COVID-19) were excluded. This exclusion aimed to focus on individuals who had the possibility of being exposed to COVID-19 for further analysis.

To ensure a comparable distribution of follow-up time between the contemporary and COVID-19 groups, the start time of follow-up was randomly assigned for the contemporary comparison groups based on the start time of follow-up for the COVID-19 group. Meanwhile, the start time of follow-up for the historical comparison group was also randomly assigned to be 3 years prior to the time of COVID-19 infection diagnosis in the COVID-19 group.

For each patient, the follow-up period was until the date of the first digestive event, mortality, or 30^th^ October 2022 for the contemporary cohort and 30^th^ October 2019 for the historical cohort, whichever occurred first.

### Definition of outcomes

Digestive outcomes were selected mainly based on evidence in prior literature and the data on digestive system disease records in the UK Biobank [2, 8, 14, 21–24]. As shown in Additional file 1: Table S2, all the outcomes were defined based on the 10th revision of the International Classification of Diseases (ICD-10). The main source of the outcome data was hospital inpatient data, supplemented by the primary care data, death register records, and self-reported medical condition codes reported at the baseline or subsequent UK Biobank assessment center visit. The outcomes included (1) functional gastrointestinal disorders (GI dysfunction): dyspepsia, irritable bowel syndrome (IBS) and constipation; (2) peptic ulcer disease (PUD): gastric ulcer, duodenal ulcer and peptic ulcer (site unspecified); (3) gastro-esophageal reflux disease (GERD); (4) inflammatory bowel disease (IBD): Crohn’s disease (CD) and ulcerative colitis (UC); (5) severe liver disease; (6) non-alcohol fatty liver disease (NAFLD); (7) gallbladder disease: cholelithiasis and cholecystitis; (8) pancreatic disease: acute pancreatitis, chronic pancreatitis, pancreatic cyst and other pancreatic diseases.

Each digestive outcome was defined as the diagnosis 30 days or more after the onset of SARS-CoV-2 infection or the index date.

### Covariates

In this study, covariates were obtained from baseline data self-reported by individuals in verbal interview and periodically updated disease diagnosis data. Pre-defined covariates were chosen in accordance with previous literature and examination of a Directed Acyclic Graph (DAG) [25, 26] (Additional file 1: Fig. S1). The covariates included sociodemographic characteristics (age, sex, ethnicity, household income, Townsend Deprivation Index), lifestyle factors (smoking status, alcohol consumption, moderate physical activity), body mass index (BMI), the number of hospital admissions 3 years before the index date (proxy of health care utilization), pre-existing comorbidities at the initiation of the study (hypertension, diabetes, heart failure, renal failure, myocardial infarction, asthma, dementia, stroke, chronic obstructive pulmonary disease [COPD]) and history of previous digestive diseases. The history of digestive diseases was defined as a compilation of diagnoses based on ICD codes (Additional file 1: Table S2) recorded before the index date, without a specific time restriction. These diagnoses were obtained from data sources including hospital inpatient records, primary care data, and self-reported medical conditions.

We calculated the number and proportion of missing data for the covariates and used multiple imputation by chained equations (MICE packages [27] in R) with predictive mean matching method on all variables, which combines regression models and nearest-neighbor matching to handle the missing data. Five imputations and 50 iterations were performed and one of the five imputations was selected randomly as the final imputed data set.

### Statistical analyses

Baseline characteristics of the COVID-19 and historical and contemporary comparison groups were reported as mean values (standard deviation) and numbers (percentages) as appropriate. Standardized mean differences (SMD) between groups were also presented.

Inverse probability weights (IPTW) were calculated for each participant to eliminate the impact of confounding factors. To test the effectiveness of weighting, we evaluated the SMD of covariates between the weighted populations. A SMD of less than 0.2 is considered to indicate adequate balance in covariates between groups. To evaluate the long-term impact of COVID-19 infection on the study outcome, Cox regression models were then constructed using the inverse probability weights and additionally adjusted for those unbalanced covariates to address the residual imbalance.

The weighted cumulative hazards plots were used to visualize the proportional hazards assumption of the Cox models constructed of the contemporary comparison group and the COVID-19 group. The comparison between the COVID-19 group and the historical comparison group is additionally examined as one of the sensitivity analyses.

To evaluate the long-term risks of digestive diseases in reinfected COVID-19 patients, we conducted a subgroup analysis through further selected individuals who experienced reinfection, defined as a positive SARS-CoV-2 test/COVID-19 diagnosis 90 days or more after the first infection (in order to reduce the likelihood of including repeated positive tests that could be attributed to the initial infection [28]). To ensure a similar distribution of follow-up time in the individuals with one SARS-CoV-2 infection and reinfection (two or more infections) groups, the start time of individuals with one SARS-CoV-2 infection were manually assigned based on the distribution of the start time of those in the reinfection group. We then constructed the non-infected comparison group of individuals with no record of a positive SARS-CoV-2 test between 30 January 2020 and 30 October 2022. We then assigned the start time to each participant in the group on the basis of the distribution of the start time in those with at least one positive SARS-CoV-2 test.

In addition, to further investigate the long-term effects of COVID-19 infection, after limiting the start time of the COVID-19 and comparison groups to 2020, sensitivity analyses were conducted by redefining the outcome as digestive diseases occurring within 6 months, between 6 months and 1 year, and 1 year to 2 years of follow-up. To evaluate whether there was any dose-dependent association of COVID-19 severity with digestive outcomes, we grouped the patients into non-hospitalized COVID, hospitalized COVID, and severe COVID-19 according to the COVID-19 severity.

Since complete data on vaccine status were unavailable, sensitivity analysis was constructed by restricting the inclusion period of the COVID-19 cohort prior to December 2020, when vaccines were still not available in the UK to avoid the effect of vaccination on the long-term risk of COVID-19. Hence, the restricted COVID-19 group was comprised of people diagnosed with COVID-19 until November 30th, 2020. For the comparisons, contemporary comparisons were limited to before November 30th, 2020, and historical comparisons were also limited to before November 30, 2017.

All analyses were performed using RStudio and R 4.2.1 software. Statistical significance was set at a two-tailed *P* value of less than 0.05.

## Results

### Baseline characteristics

Additional file 1: Fig. S2 demonstrates the criteria used for selecting study cohorts. The COVID-19 group consisted of 112,311 individuals, the contemporary comparison group had 359,671 individuals, and the historical comparison group had 370,979 individuals. The median follow-up time was 254 (interquartile range [IQR] 184–366) days for the COVID-19 group, 254 (IQR 184–366) days for the contemporary comparison group, and 254 (IQR 184–367) days for the historical comparison group. The distribution of follow-up time is provided in Additional file 1: Fig. S3. The number and percentage of missing covariates are shown in Additional file 1: Table S3.

### Risks of digestive diseases in long COVID

#### COVID-19 group versus contemporary comparison group

The baseline characteristics of the study population before and after weighting were presented in Table 1 and Additional file 1: Table S4. After weighting, all characteristics were balanced between the groups.

**Table 1:** Baseline characteristics of COVID-19 group and contemporary comparison after weighting

Characteristics	COVID-19 group (*n* = 112,311)	Contemporary comparison (*n* = 359 671)	SMD
Age, mean (SD), years	56.2 (8.1)	56.2 (8.1)	0.002
Sex, female, *n* (%)	61,546 (54.8)	198,538 (55.2)	0.008
Ethnicity, White, *n* (%)	106,134 (94.5)	339,889 (94.5)	0.001
Household income			0.004
<18,000, *n* (%)	24,259 (21.6)	78,049 (21.7)	
18,000–30,999, *n* (%)	28,527 (25.4)	91,716 (25.5)	
31,000–51,999, *n* (%)	29,650 (26.4)	94,953 (26.4)	
52,000–100,000, *n* (%)	23,473 (20.9)	74,452 (20.7)	
>100,000, *n* (%)	6514 (5.8)	20,501 (5.7)	
Deprivation index, mean (SD)	−1.3 (3.0)	−1.3 (3.1)	0.002
BMI, mean (SD), kg/m^2^	27.4 (4.7)	27.4 (4.8)	0.001
Alcohol consumption			0.005
Daily or almost daily, *n* (%)	22,799 (20.3)	72,654 (20.2)	
Three or four times a week, *n* (%)	26 281 (23.4)	83,803 (23.3)	
Once or twice a week, *n* (%)	29,089 (25.9)	93,514 (26.0)	
One to three times a month, *n* (%)	12,579 (11.2)	40,283 (11.2)	
Special occasions only or never, *n* (%)	12,691 (11.3)	41,002 (11.4)	
Never, *n* (%)	8873 (7.9)	28,414 (7.9)	
Smoking status			0.002
Never smoker,* n* (%)	62,557 (55.7)	200,696 (55.8)	
Previous smoker, *n* (%)	38,523 (34.3)	123,007 (34.2)	
Current smoker, *n* (%)	11,231 (10.0)	35,967 (10.0)	
Physical activity, mean (SD), MET minutes/week	2644.0 (2701.5)	2649.9 (2708.2)	0.002
Comorbidities			
Hypertension, *n* (%)	40,544 (36.1)	129,841 (36.1)	0.001
Diabetes, *n* (%)	8536 (7.6)	27,335 (7.6)	0.001
Renal failure, *n* (%)	4717 (4.2)	15,106 (4.2)	0.001
Myocardial infarction, *n* (%)	4829 (4.3)	15,466 (4.3)	0.001
Stroke, *n* (%)	2920 (2.6)	9351 (2.6)	0.002
COPD, *n* (%)	4605 (4.1)	14,747 (4.1)	0.004
Asthma, *n* (%)	15,611 (13.9)	49,635 (13.8)	0.002
Heart failure, *n* (%)	2471 (2.2)	7913 (2.2)	0.002
Dementia, *n* (%)	786 (0.7)	2518 (0.7)	0.002
Recent hospital admissions, mean (SD)	0.9 (1.7)	0.9 (1.8)	0.009
History of previous digestive diseases, *n* (%)	36,838 (32.8)	117,612 (32.7)	0.003

*SMD* standard mean difference, *BMI* body mass index, *MET* metabolic equivalent of task, *COPD* chronic obstructive pulmonary disease, *SD* standard deviation

**Fig. 1:**
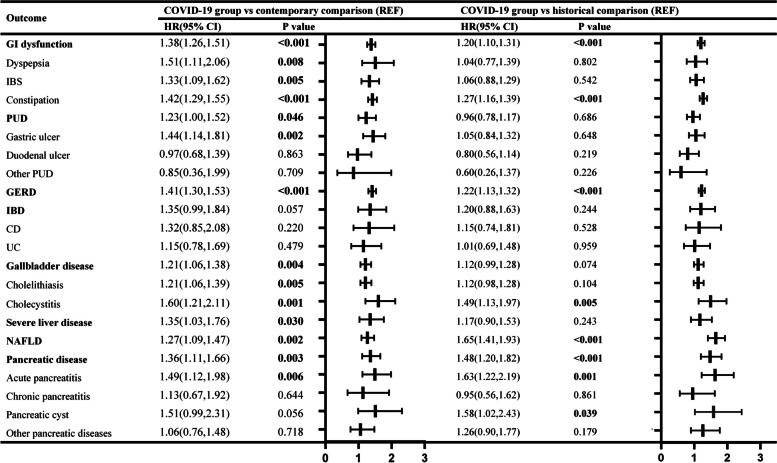
Hazard ratio of digestive outcomes in the COVID-19 group compared to the contemporary and historical comparisons. HR: hazard ratios; CI: confidence interval; Outcomes were ascertained 30 days after the COVID-19-positive test until the end of follow-up. Weighted HRs after IPTW and 95% CIs are presented

**Fig. 2:**
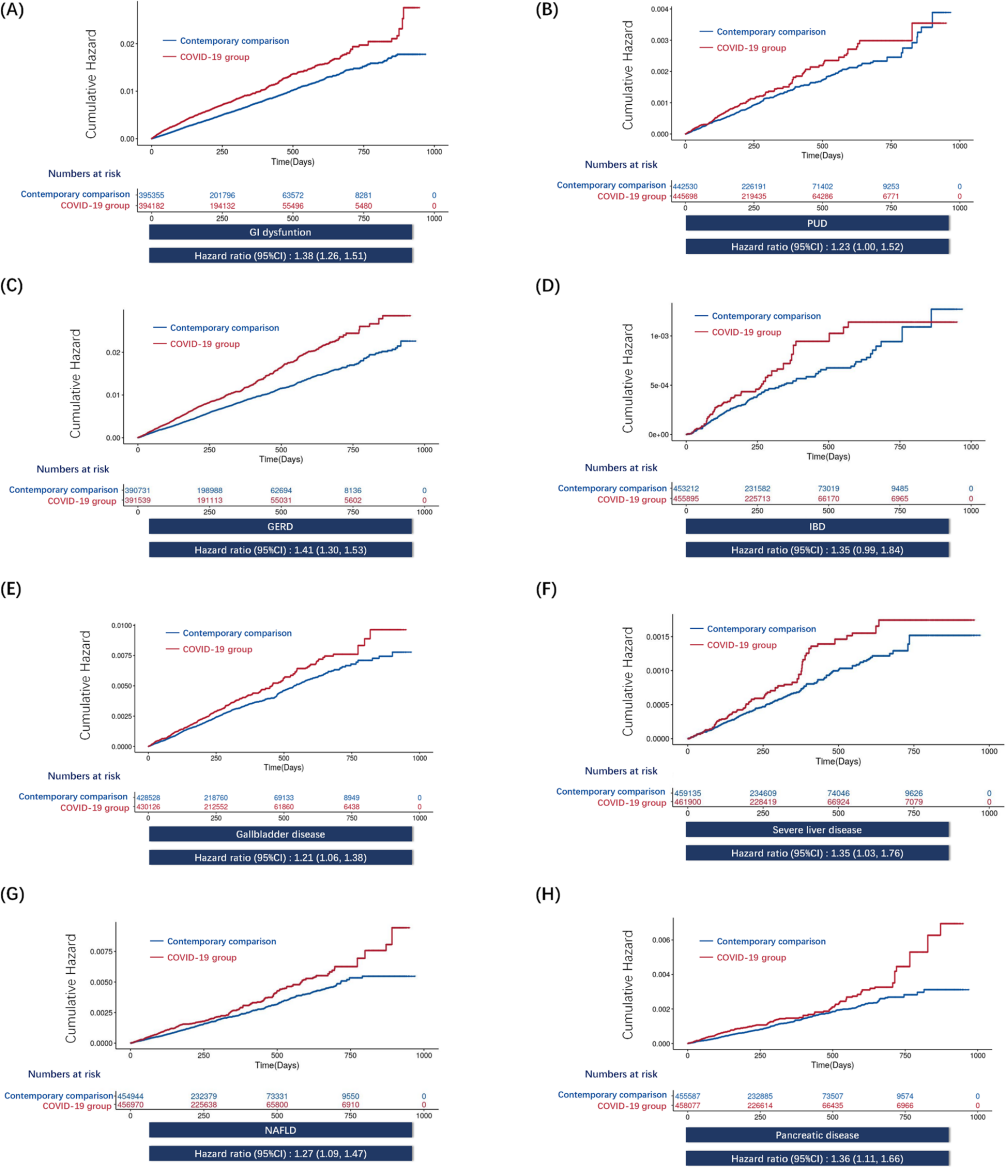
IPTW-weighted cumulative hazards plots of the COVID-19 group and contemporary comparison in digestive outcomes. The cumulative hazard and weighted hazard ratios for GI dysfunction **A**, PUD **B**, GERD **C**, IBD **D**, gallbladder disease **E**, severe liver diseases **F**, NAFLD **G**, and pancreatic disease **H** in COVID-19 group as compared with contemporary comparison are illustrated. 95% confidence intervals are shown in parentheses

Figure 1 provided the risks of digestive outcomes in these groups. Compared to the contemporary comparison group, people who survived the first 30 days of COVID-19 showed an increased risk of GI dysfunction (HR 1.38 (95% confidence interval [CI] 1.26 to 1.51), *P* < 0.001); PUD (HR 1.23 (1.00 to 1.52), *P* = 0.046); GERD (HR 1.41 (1.30 to 1.53), *P* < 0.001); gallbladder disease (HR 1.21 (1.06 to 1.38), *P* = 0.004); severe liver disease (HR 1.35 (1.03 to 1.76), *P* = 0.030); NAFLD (HR 1.27 (1.09 to 1.47), *P* = 0.002); and pancreatic disease (HR 1.36 (1.11 to 1.66), *P* = 0.003). On the contrary, 30-day survivors of COVID-19 exhibited no increased risks of IBD (HR 1.35 (0.99 to 1.84), *P* = 0.054). The weighted cumulative hazard plots are shown in Fig. 2.

Additional file 1: Table S5 shows the results of the COVID-19 group compared with the contemporary comparison group at different follow-up times. We found persistent increased risks of GI dysfunction and GERD in the COVID-19 group after 1 year of follow-up. In contrast, the risk of gallbladder disease was only observed within 6 months of post-infection. There was no rise in the risk of other digestive diseases after a follow-up period of 6 months.

We further examined the risks of digestive outcomes by the severity of COVID-19 infection during the acute phase (non-hospitalized [*n* = 104,201], hospitalized [*n* = 7523], and severe COVID-19 [*n* = 588]); Baseline characteristics of these groups before and after weighting were provided in Additional file 1: Table S6, S7. Most covariates were well balanced after the application of weights. Then we adjusted for those covariates that remained unbalanced in the IPTW-weighted Cox models. In comparison to the contemporary comparison group, the risks of GI dysfunction, PUD, GERD, and NAFLD were evident even among non-hospitalized COVID-19 patients (Additional file 1: Fig. S4). A positive correlation was observed between the severity of acute COVID infection and the risk of developing GERD with increasing risk from non-hospitalized, hospitalized to severe COVID.

In the subgroup analysis on the risks of digestive outcomes associated with SARS-CoV-2 reinfection, the cohort consisted of the non-infected group (*n* = 359,671), single SARS-CoV-2 infection group (*n* = 107,950) and reinfected group (two or more infections) (*n* = 4361). Additional file 1: Table S8, S9 showed the baseline characteristics of these groups before and after weighting. Compared with the non-infected comparisons, the risks of pancreatic diseases were higher among reinfected patients (HR 5.40 (2.22 to 13.15), *P* < 0.001) than that among patients with a single infection (HR 1.44 (1.11 to 1.88), *P* = 0.007) (Additional file 1: Table S10). For head-to-head comparison, reinfected patients were at an even higher risk for pancreatic diseases (HR 2.57 (1.23 to 5.38), *P* = 0.012) compared with those who only experienced a single infection (Additional file 1: Table S11).

In the sensitivity analysis, the COVID-19 group consisted of 8,431 individuals when restricting to the period before vaccination was available in the UK. Additional file 1: Table S12, S13 demonstrated baseline characteristics of the COVID-19 group and contemporary comparisons before and after weighting, indicating all covariates were well balanced. The results were basically in agreement with the main analyses (Additional file 1: Table S14).

Additionally, we conducted analyses by pooling estimates across all five imputed datasets (Additional file 1: Table S15). This result was consistent with our primary analyses when using one imputed dataset.

#### COVID-19 group versus contemporary comparison group in subgroup analyses

In parallel, we examined the risks of digestive outcomes in the COVID-19 group compared to contemporary comparisons in pre-specified subgroups. Subgroup analyses found consistent results that the risks of GI dysfunction and GERD were evident in all subgroups (Additional file 1: Table S16).

As shown in Additional file 1: Table S17, no sex-based differences in digestive diseases between COVID-19 patients and comparison groups were observed in subgroup analyses by sex.

#### COVID-19 group versus historical comparison group

We assessed the reliability of our study design by using a historical comparison group, from a period preceding the COVID-19 pandemic, as the reference category. Baseline characteristics before and after weighting in Additional file 1: Table S18-S23, suggested that most covariates were balanced after weighting. Unbalanced covariates were further adjusted. The trend of risks was similar with the results of analyses using the contemporary comparison in comparisons of the COVID-19 group (Fig. 1; Additional file 1: Table S14-S17, S24).

## Discussion

Our research demonstrated a significant association between COVID-19 and heightened risks of GI dysfunction, GERD, and other digestive system ailments in the long term. The risks of GI dysfunction, PUD, GERD, and NAFLD were evident even in non-hospitalized COVID-19 patients. In addition, the risks of GI dysfunction and GERD did not decrease after 1-year follow-up, revealing the long-term effect of COVID-19 and the risks of digestive disorders. The findings contribute to a better understanding of the systemic impact of COVID-19, as well as emphasizing the importance of prevention and early intervention of these digestive system sequela.

In this study, we have identified several key findings. First, our study indicated that COVID-19 was significantly associated with an elevated long-term risk of developing various digestive system diseases. This is consistent with the previous study by Xu et al. based on the US Veteran Health Administration (VHA) database [14]. Considering the non-negligible selection bias of the US VHA cohort with nearly 90% male of veteran participants, the result of our study exhibits a high degree of generalizability, owing to the inclusion of both male and female in the UK Biobank cohort. Besides, in an attempt to minimize the impact of vaccination, a confounding factor has not been fully addressed in previous research, we carried out subgroup analyses exclusively within the period prior to vaccination, which provides additional evidence for the reliability of our findings.

Secondly, we found that even in people with mild COVID-19 symptoms who did not receive hospitalization treatment, the risks of GI dysfunction, PUD, GERD, and NAFLD were evident, while the risks of severe liver disease, IBD, and biliopancreatic diseases were not. Besides, the severity of COVID-19 was associated with an increased risk of developing GERD, while no association was observed with the risks of GI dysfunction, PUD, IBD, hepatobiliary and pancreatic diseases. This is different from the results of Xu et al.’s study [14], which found that the risks of certain biliopancreatic diseases (acute pancreatitis and cholangitis) and biliary function tests were evident among non-hospitalized COVID-19 patients; the risks of GI dysfunction related disorders (IBS and constipation), PUD, acute pancreatitis, as well as hepatobiliary function tests, increased stepwise according to the severity of COVID-19. The reason underlying the discrepant results in these digestive system outcomes with sex-dependent differences might be partly explained by the sex bias of the US VHA cohort as mentioned previously [29–33]. Given that people with mild symptoms take up over 95% of the COVID-19 population, even a small incidence rate can result in a large number of people being affected. This underscores the significance of ensuring that healthcare systems are equipped to provide appropriate care to this population of mild cases, as well as varying degrees of COVID-19 severity.

Thirdly, we investigated the differences in risks across different time periods, as well as the prolonged impacts of COVID-19 on digestive system diseases. Our study revealed that the risks of GI dysfunction and GERD persisted even after 1-year follow-up, indicating the long-term effect of COVID-19 on the risks of specific digestive disorders. In contrast, our study demonstrated only the short-term risks of developing gallbladder disease in post-COVID with 6 months. Other digestive diseases did not show an increased risk after a follow-up of 6 months. Due to the long follow-up (up to 2 years and a half), our study meaningfully extended previous findings constrained by the short follow-up (typically less than 12 months) [12, 34, 35] and lack of distinction between different follow-up time periods [14]. This sheds light on the importance of enhancing our comprehension of the varying risks of digestive outcomes in COVID-19 patients across different time periods to develop appropriate care strategies during the post-acute phase and long-term clinical follow-up post-recovery.

Fourthly, our study showed a significant increase of the long-term risk of developing pancreatic diseases among individuals who suffered SARS-CoV-2 reinfection. The finding emphasizes the importance of continued vigilance in preventing reinfection to safeguard public health and mitigate the potential burden of SARS-CoV-2 reinfection in the future. By identifying specific diseases with higher long-term risks in reinfected individuals, heightened awareness and tailored prevention and treatment strategies can be implemented in a targeted manner for those at risk.

The mechanisms behind the associations between COVID-19 and digestive diseases are not yet fully understood, but several possibilities have been suggested. One possibility is the fecal-oral transmission of the virus, leading to viral infection of the digestive tract [36]. After the acute phase, the virus infection usually triggers IBS which causes long-term functional gastrointestinal disorders of the digestive tract [37].

Additionally, interactions between the SARS-CoV-2 spike protein and the expression of the angiotensin-converting enzyme 2 (ACE2) receptor on the digestive tract might also be involved in the progression of digestive diseases among COVID-19 patients. ACE2 is the major receptor for SARS-CoV-2 spike proteins during infection [38]. In fact, the epithelium of the gastrointestinal tract expresses a higher level of ACE2 than the lung [38], which makes it highly susceptible to SARS-CoV-2 infection [39]. ACE2 also expresses in the biliary tract and pancreas, which may contribute to the increased risk of gallbladder and pancreas diseases after COVID-19 [40].

Another potential mechanism is that COVID-19 infection has been linked to significant elevations in inflammatory markers and cytokines, including interleukin-1 (IL-1), IL-6, and tumor necrosis factor-alpha (TNF-α) [41]. These elevated levels play a role in the infiltration of immune cells in the gastrointestinal tract, leading to the subsequent initiation of hepatocellular cholestasis through the inhibition of hepatobiliary uptake and excretory mechanisms [42, 43].

## Strengths

Our study has several strengths that contribute to the validity and reliability of our findings. Firstly, our study had a long-term follow-up period of up to two and a half years, which allowed us to analyze the sustained effects of COVID-19 on digestive system diseases. This extended follow-up period adds further value to our study, as it provides insight into the long-term implications of COVID-19 on digestive health. Secondly, by using the UK Biobank, a nationwide cohort including both female and male, the results of our study were of high generalizability compared to the only large-scale study of US VHA. Thirdly, to enhance the robustness of the results, we employed two comparison groups including a contemporary and a historical cohort, allowing the analysis to be conducted more comprehensively. Furthermore, we conducted the subgroup analyses restricting to the period before vaccination was available to eliminate the impact of vaccination, which has been understudied in prior researches. Fourth, we investigated the dose-response relationship of patients of COVID-19 severity and digestive diseases.

## Limitations

Although our study provides important insights into the association between COVID-19 and digestive system diseases, it is not without limitations. Firstly, our study was conducted predominantly on a European population and there also exists healthy volunteer selection bias, as individuals enrolled in the UK Biobank exhibited better health conditions compared to the general population [20]; further studies in other ethnicities are warranted to confirm our results. Secondly, being an observational study, we cannot establish a causal relationship between COVID-19 and the long-term risk of digestive system diseases. However, we observed dose-response relationship between the severity of COVID-19 and the risk of GERD, which to some extent suggested a possibility for causality. Thirdly, the potential inclusion of individuals with undiagnosed or untested COVID-19 infections in the comparison group, rather than the COVID-19 group, introduces the possibility of misclassification of exposure. However, this likely biases estimates towards the null. Fourthly, there may be selection bias in our study. The loss of follow-up during the first month after infection could be influenced by both COVID-19 infection (e.g., its severity) and subsequent health outcomes, leading to differences in the characteristics of the final population included in the analysis compared to those of the original population under study. Meanwhile, for the contemporary comparison groups, confounding factors during the pandemic (e.g., policy interventions, behavioral changes) might bias the results [44]; for the historical comparison group, the comparability might be reduced by temporal changes [45].

## Conclusion

In conclusion, our study contributes to the growing body of evidence on the long-term impact of COVID-19 on the digestive system. Specifically, there are continuing risks of GI dysfunction and GERD requiring long-term follow-up and further attention. Our findings highlight the need for long-term care and management of COVID-19 patients to monitor potential post-acute complications of the digestive system. Meanwhile, comprehensive awareness of the varying risks of digestive outcomes in COVID-19 patients over time is significant to develop appropriate follow-up strategies.

### Supplementary Information

**Additional file 1:** **Figure S1.** Directed Acyclic Graphs (DAG) for covariate selection. **Figure S2.** Flow chart of eligible participants’ selection. **Figure S3.** Distribution of follow-up time in the contemporary cohort (A) and the historical cohort (B). **Figure S4.** Hazard ratio of digestive outcomes in COVID-19 group and the contemporary comparison by severity of COVID-19. **Table S1.** Respiratory support treatments definition. **Table S2.** Outcome ascertainment. **Table S3.** The numbers (percentages) of participants with missing covariates. **Table S4.** Baseline characteristics of COVID-19 group and contemporary comparisons before weighting. **Table S5.** Hazard ratio of digestive outcomes in COVID-19 group and the contemporary comparison at different follow-up times. **Table S6.** Baseline characteristics of COVID-19, contemporary comparisons by severity of COVID-19 before weighting. **Table S7.** Baseline characteristics of COVID-19, contemporary comparisons by severity of COVID-19 after weighting. **Table S8.** Baseline characteristics of COVID-19 group and contemporary comparisons by status of SARS-CoV reinfection before weighting. **Table S9.** Baseline characteristics of COVID-19 group and contemporary comparisons by severity of SARS-CoV reinfection after weighting. **Table S10.** Hazard ratio of digestive outcomes in the reinfected group, single SARS-CoV-2 infection group, and non-infected comparisons. **Table S11.** Hazard ratio of digestive outcomes in reinfected group and single SARS-CoV-2 infection group in head-to-head comparison. **Table S12.** Baseline characteristics of COVID-19 group and contemporary comparisons in the sensitive analysis restricting to the period before vaccination was available before weighting. **Table S13.** Baseline characteristics of COVID-19 group and contemporary comparisons in the sensitive analysis restricting to the period before vaccination was available after weighting. **Table S14.** Hazard ratio of digestive outcomes in COVID-19 group and contemporary and historical comparisons in subgroups in the sensitive analysis restricting to the period before vaccination was available. **Table S15.** Hazard ratio of digestive outcomes in COVID-19 group compared to the contemporary and historical comparisons by pooling estimates across all five imputed datasets. **Table S16.** Hazard ratio of digestive outcomes compared with contemporary and historical comparisons in subgroups. **Table S17.** Hazard ratio of digestive outcomes in COVID-19 group, the contemporary and historical comparison by sex. **Table S18.** Baseline characteristics of COVID-19 group and historical comparisons before weighting. **Table S19.** Baseline characteristics of COVID-19 group and historical comparisons after weighting. **Table S20.** Baseline characteristics of COVID-19 group and historical comparisons by severity of COVID-19 before weighting. **Table S21.** Baseline characteristics of COVID-19 group and historical comparisons by severity of COVID-19 after weighting. **Table S22.** Baseline characteristics of COVID-19 group and historical comparisons in the sensitive analysis restricting to the period before vaccination was available before weighting. **Table S23.** Baseline characteristics of COVID-19 group and historical comparisons in the sensitive analysis restricting to the period before vaccination was available after weighting. **Table S24.** Hazard ratio of digestive outcomes in COVID-19 group and the historical comparison by severity of COVID-19.

## Data Availability

The UK Biobank data are available on application to the UK Biobank (http://www.ukbiobank.ac.uk/register-apply).

## Abbreviations

ACE2: Angiotensin-converting enzyme 2
BMI: Body mass index
CD: Crohn’s disease
CI: Confidence intervals
COPD: Chronic obstructive pulmonary disease
COVID-19: Coronavirus disease 2019
DAG: Directed acyclic graph
GERD: Gastroesophageal reflux disease
GI: Gastrointestinal
HR: Hazard ratios
IBD: Inflammatory bowel disease
IBS: Irritable bowel syndrome
IPTW: Inverse probability treatment weighting
IQR: Interquartile range
NAFLD: Non-alcohol fatty liver disease
PUD: Peptic ulcer disease
SARS-CoV-2: Severe acute respiratory syndrome coronavirus 2
SD: Standard deviation
SMD: Standardized mean difference
UC: Ulcerative colitis
