# Supplementary material for: Risks of digestive diseases in long COVID: evidence from a population-based cohort study
Source: BMC Med. 2024 Jan 10;22:14. doi: 10.1186/s12916-023-03236-4 (PMC10777515; doi:10.1186/s12916-023-03236-4)
Supplement: Supplementary file 1 — Additional file 1: Figure S1. Directed Acyclic Graphs (DAG) for covariate selection. Figure S2. Flow chart of eligible participants’ selection. Figure S3. Distribution of follow-up time in the contemporary cohort (A) and the historical cohort (B). Figure S4. Hazard ratio of digestive outcomes in COVID-19 group and the contemporary comparison by severity of COVID-19. Table S1. Respiratory support treatments definition. Table S2. Outcome ascertainment. Table S3. The numbers (percentages) of participants with missing covariates. Table S4. Baseline characteristics of COVID-19 group and contemporary comparisons before weighting. Table S5. Hazard ratio of digestive outcomes in COVID-19 group and the contemporary comparison at different follow-up times. Table S6. Baseline characteristics of COVID-19, contemporary comparisons by severity of COVID-19 before weighting. Table S7. Baseline characteristics of COVID-19, contemporary comparisons by severity of COVID-19 after weighting. Table S8. Baseline characteristics of COVID-19 group and contemporary comparisons by status of SARS-CoV reinfection before weighting. Table S9. Baseline characteristics of COVID-19 group and contemporary comparisons by severity of SARS-CoV reinfection after weighting. Table S10. Hazard ratio of digestive outcomes in the reinfected group, single SARS-CoV-2 infection group, and non-infected comparisons. Table S11. Hazard ratio of digestive outcomes in reinfected group and single SARS-CoV-2 infection group in head-to-head comparison. Table S12. Baseline characteristics of COVID-19 group and contemporary comparisons in the sensitive analysis restricting to the period before vaccination was available before weighting. Table S13. Baseline characteristics of COVID-19 group and contemporary comparisons in the sensitive analysis restricting to the period before vaccination was available after weighting. Table S14. Hazard ratio of digestive outcomes in COVID-19 group and contemporary and historical comparison [file 12916_2023_3236_MOESM1_ESM.docx]

Figure S1. [Directed Acyclic Graphs (DAG) for covariate selection.](https://www.ncbi.nlm.nih.gov/pmc/articles/PMC7532091/bin/12889_2020_9552_MOESM3_ESM.pdf)


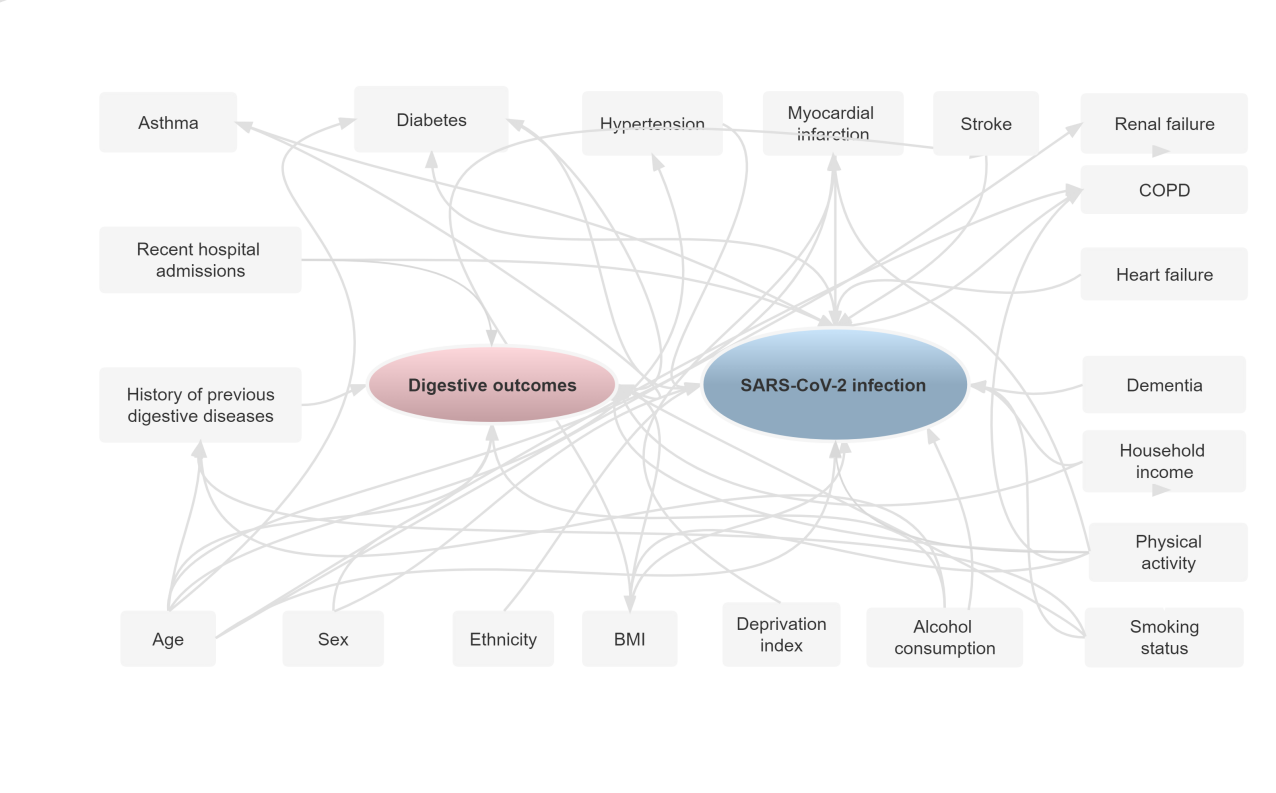


DAG showing the relationships between the study variables.

Figure S2. Flow chart of eligible participants’ selection.


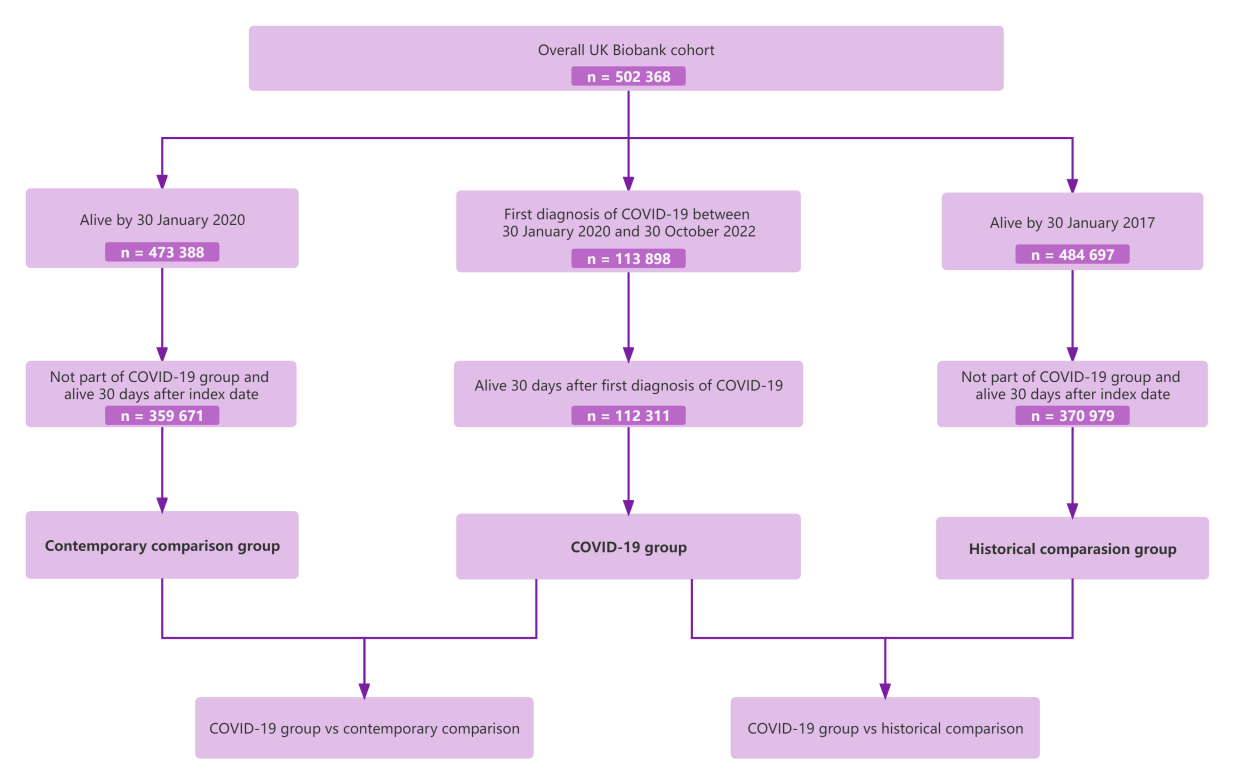


Figure S3. Distribution of follow-up time in the contemporary cohort (A) and the historical cohort (B).


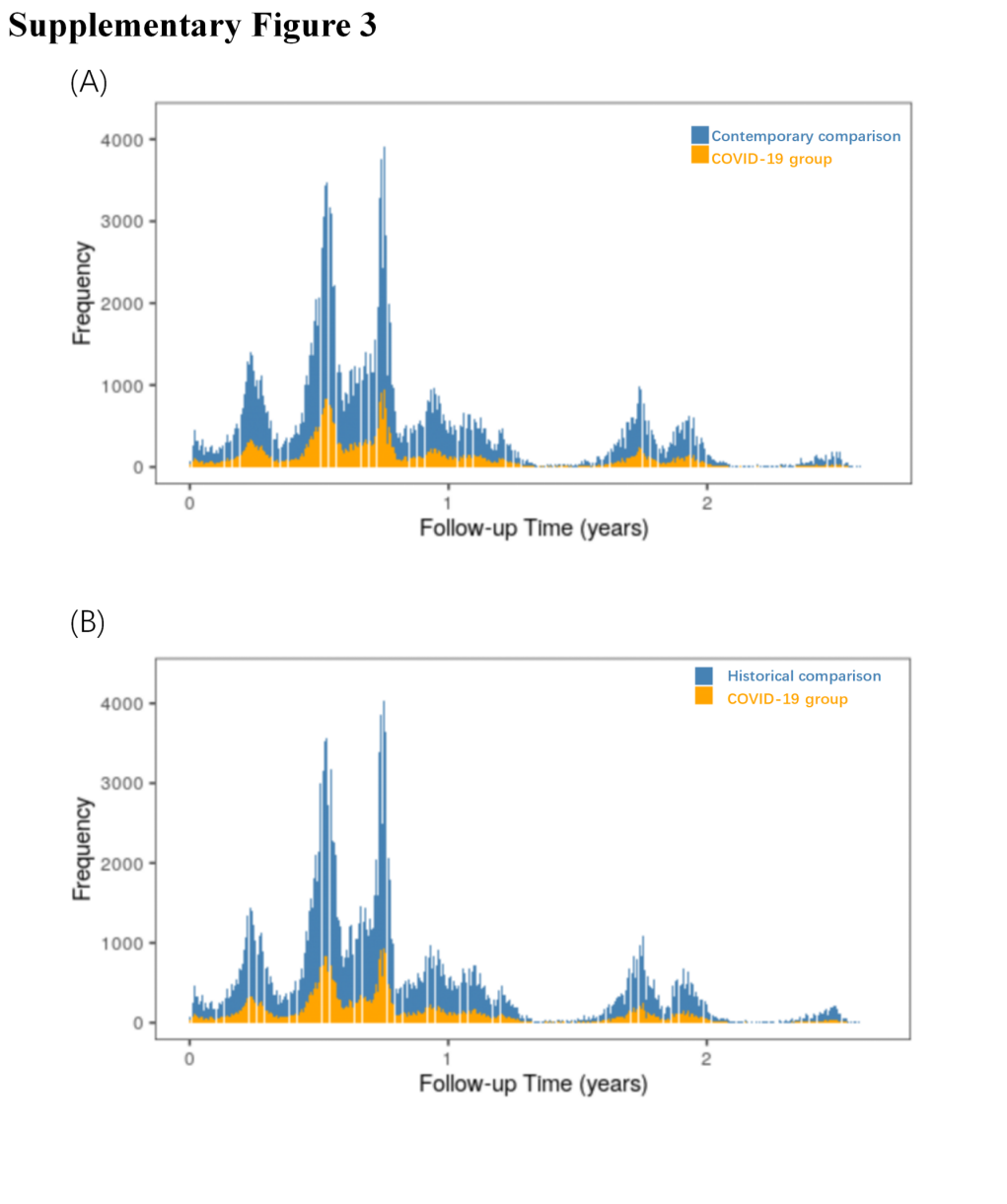


Figure S4. Hazard ratio of digestive outcomes in COVID-19 group and the contemporary comparison by severity of COVID-19.


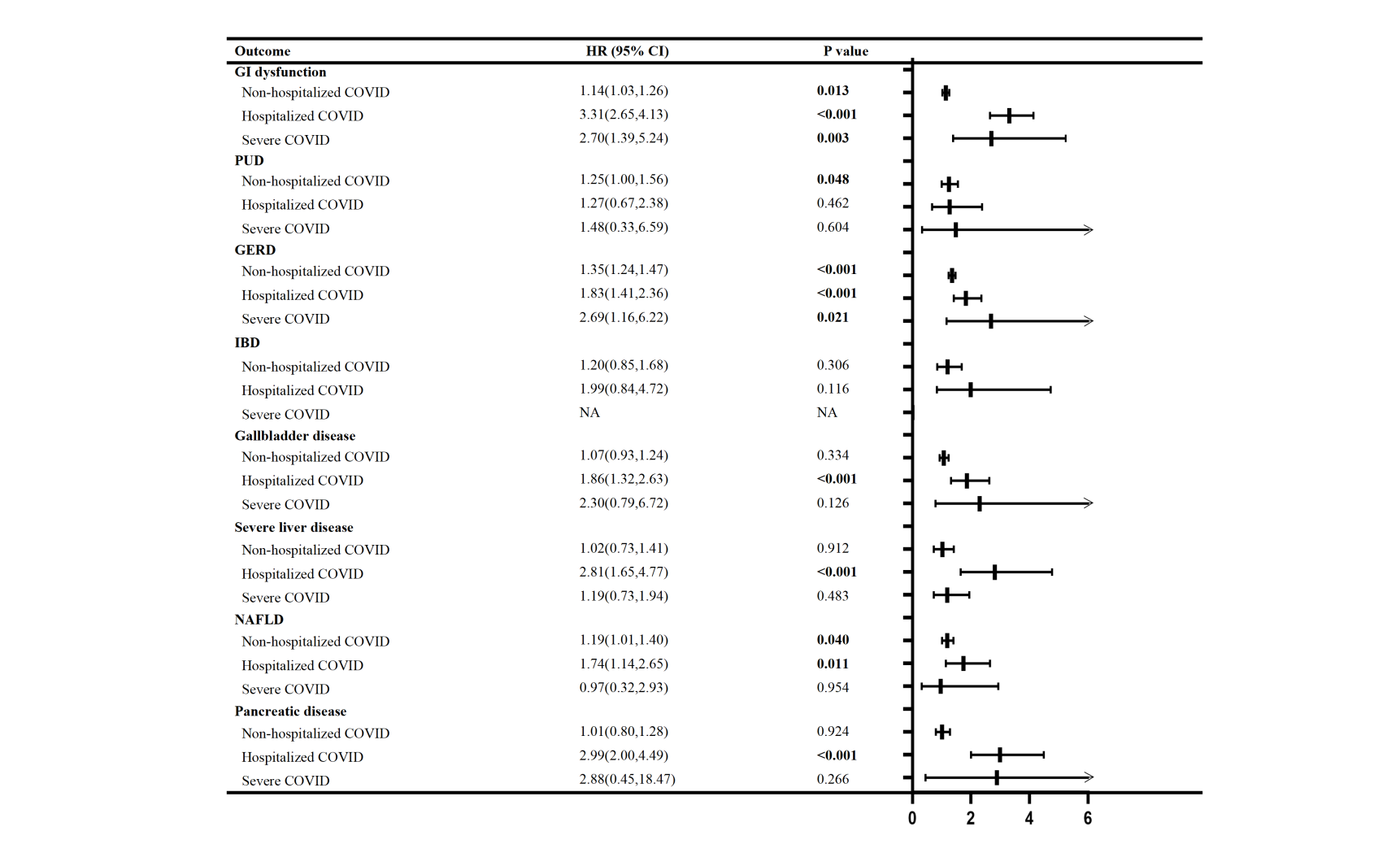


HR: hazard ratios; CI: confidence interval;

Outcomes were ascertained 30 days after the COVID-19-positive test until the end of follow-up. Weighted HRs after IPTW and 95% CIs are presented. Table S1. Respiratory support treatments definition.

| Outcome | OPCS-4-codes |
| --- | --- |
| Invasive ventilation | E85.1 |
| Continuous positive airway pressure | E85.6 |
| Non-invasive ventilation | E85.2 |
| Unspecified oxygen therapy | X52.9 |
| Other specified oxygen therapy | X52.8 |
| Other specified ventilation support | E85.8 |
| Unspecified ventilation support | E85.9 |
| Other specified oxygen therapy support | E87.8 |
| Unspecified oxygen therapy support | E87.9 |

Table S2. Outcome ascertainment.

| Outcome | ICD-10 |
| --- | --- |
| GI dysfunction |  |
| Dyspepsia | K30 |
| Irritable bowel syndrome | K58 |
| Constipation | K590 |
| PUD |  |
| Gastric ulcer | K25 |
| Duodenal ulcer | K26 |
| Other PUD | K27 |
| GERD | K21 |
| IBD |  |
| CD | K50 |
| UC | K51 |
| Gallbladder disease |  |
| Cholelithiasis | K80 |
| Cholecystitis | K81 |
| Severe liver disease | K703, K704, K721, K729, K741, K742, K746, I850, I859, K766, K767 |
| NAFLD | K758, K759, K760 |
| Pancreatic disease |  |
| Acute pancreatitis | K85 |
| Chronic pancreatitis | K861 |
| Pancreatic cyst | K862, K863 |
| Other pancreatic diseases | K868, K869 |

GI, gastrointestinal; IBS, irritable bowel syndrome; PUD, peptic ulcer disease; IBD, inflammatory bowel disease; CD, Crohn’s disease; UC, ulcerative colitis; NAFLD, non-alcohol fatty liver disease

Table S3. The numbers (percentages) of participants with missing covariates

| Covariates | N | % |
| --- | --- | --- |
| Ethnicity | 2 777 | 0.55% |
| Household income | 6 012 | 1.20% |
| Deprivation index | 624 | 0.12% |
| BMI | 3 105 | 0.62% |
| Alcohol consumption | 1 501 | 0.30% |
| Smoking status | 2 949 | 0.59% |
| Physical activity | 100 103 | 19.93% |

BMI: body mass index

Table S4. Baseline characteristics of COVID-19 group and contemporary comparisons before weighting

| Characteristics | COVID-19 group  (n = 112 311) | Contemporary comparisons  (n = 359 671) | SMD |
| --- | --- | --- | --- |
| Age, mean(SD), years | 54.43(8.19) | 56.79(7.97) | 0.293 |
| Sex, female, n(%) | 61 495(54.8) | 199 368(55.4) | 0.014 |
| Ethnicity, White, n(%) | 106 315(94.7) | 339 676(94.4) | 0.010 |
| Household income |  |  | 0.189 |
| <18 000, n(%) | 19 465(17.3) | 82 983(23.1) |  |
| 18 000-30 999, n(%) | 26 102(23.2) | 94 121(26.2) |  |
| 31 000-51 999, n(%) | 31 853(28.4) | 92 659(25.8) |  |
| 52 000-100 000, n(%) | 27 297(24.3) | 70 470(19.6) |  |
| >100 000, n(%) | 7 594(6.8) | 19 438(5.4) |  |
| Deprivation index, mean(SD) | -1.38(3.00) | -1.32(3.09) | 0.023 |
| BMI, mean(SD), kg/m^2^ | 27.47(4.82) | 27.36(4.74) | 0.025 |
| Alcohol consumption |  |  | 0.099 |
| Daily or almost daily, n(%) | 21 925(19.5) | 73 209(20.4) |  |
| Three or four times a week, n(%) | 28 027(25.0) | 81 886(22.8) |  |
| Once or twice a week, n(%) | 30 734(27.4) | 91 957(25.6) |  |
| One to three times a month, n(%) | 12 790(11.4) | 40 314(11.2) |  |
| Special occasions only or never, n(%) | 11 298(10.1) | 42 710(11.9) |  |
| Never, n(%) | 7 537(6.7) | 29 595(8.2) |  |
| Smoking status |  |  | 0.053 |
| Never smoker, n(%) | 63 505(56.5) | 199 721(55.5) |  |
| Previous smoker, n(%) | 38 875(34.6) | 122 502(34.1) |  |
| Current smoker, n(%) | 9 931(8.8) | 37 448(10.4) |  |
| Physical activity, mean(SD), MET minutes/week | 2536.46(2603.69) | 2686.42(2739.02) | 0.056 |
| Comorbidities |  |  |  |
| Hypertension, n(%) | 38 023(33.9) | 132 105(36.7) | 0.060 |
| Diabetes, n(%) | 8 384(7.5) | 27 531(7.7) | 0.007 |
| Renal failure, n(%) | 4 646(4.1) | 15 075(4.2) | 0.003 |
| Myocardial infarction, n(%) | 4 557(4.1) | 15 720(4.4) | 0.016 |
| Stroke, n(%) | 2 706(2.4) | 9 577(2.7) | 0.016 |
| COPD, n(%) | 4 377(3.9) | 14 699(4.1) | 0.010 |
| Asthma, n(%) | 17 231(15.3) | 48 079(13.4) | 0.056 |
| Heart failure, n(%) | 2 356(2.1) | 7888(2.2) | 0.007 |
| Dementia, n(%) | 1 060(0.9) | 2 322(0.6) | 0.034 |
| Recent hospital admissions, mean(SD) | 1.0(1.9) | 0.9(1.7) | 0.076 |
| History of previous digestive diseases, n(%) | 37 375(33.3) | 116 937(32.5) | 0.016 |

SMD: standard mean difference; BMI: body mass index; MET: metabolic equivalent of task; COPD: chronic obstructive pulmonary disease; SD: standard deviation

Table S5. Hazard ratio of digestive outcomes in COVID-19 group and the contemporary comparison at different follow-up times

| Outcome | 0 to 6-month follow-up | | 6 to 12-month follow-up | | 12 to 24-month follow up | |
| --- | --- | --- | --- | --- | --- | --- |
|  | HR (95% CI) | P value | HR (95% CI) | P value | HR (95% CI) | P value |
| GI dysfunction | 1.96(1.48,2.60) | **<0.001** | 1.20(0.84,1.71) | 0.322 | 1.35(1.04,1.75) | **0.023** |
| PUD | 1.08(0.51,2.28) | 0.838 | 1.04(0.37,2.93) | 0.942 | 1.49(0.79,2.82) | 0.218 |
| GERD | 1.86(1.38,2.52) | **<0.001** | 1.36(0.97,1.91) | 0.072 | 1.64(1.30,2.07) | **<0.001** |
| IBD | 1.48(0.56,3.90) | 0.424 | 2.26(0.89,5.75) | 0.087 | 0.85(0.24,3.00) | 0.797 |
| Gallbladder disease | 1.86(1.26,2.76) | **0.002** | 1.48(0.93,2.35) | 0.095 | 1.29(0.88,1.89) | 0.188 |
| Severe liver disease | 1.64(0.74,3.60) | 0.221 | 1.56(0.57,4.26) | 0.385 | 1.49(0.64,3.46) | 0.359 |
| NAFLD | 1.63(0.95,2.77) | 0.074 | 1.47(0.87,2.51) | 0.153 | 1.40(0.93,2.10) | 0.103 |
| Pancreatic disease | 1.49(0.77,2.88) | 0.237 | 1.22(0.54,2.79) | 0.633 | 1.63(0.99,2.68) | 0.057 |

GI, gastrointestinal;GERD, gastroesophageal reflux disease; PUD, peptic ulcer disease; IBD, inflammatory bowel disease; NAFLD, non-alcohol fatty liver disease;

HR: hazard ratio; CI: confidence interval;

Weighted HRs after IPTW and 95% CIs are presented.

Table S6. Baseline characteristics of COVID-19, contemporary comparisons by severity of COVID-19 before weighting

| Characteristics | Non-hospitalized COVID  (n= 104 201) | Hospitalized COVID  (n= 7 523) | Severe COVID  (n= 588) | Contemporary comparisons  (n = 359 671) | SMD | | |
| --- | --- | --- | --- | --- | --- | --- | --- |
|  |  |  |  |  | Non-hospitalized COVID and contemporary comparisons | Hospitalized COVID and contemporary comparisons | Severe COVID and contemporary comparisons |
| Age, mean(SD), years | 54.02(8.09) | 59.84(7.55) | 57.78(7.99) | 56.79(7.97) | 0.346 | 0.393 | 0.124 |
| Sex, female, n(%) | 57 959(55.6) | 3 324(44.2) | 212(36.1) | 199 368(55.4) | 0.004 | 0.226 | 0.397 |
| Ethnicity, White, n(%) | 98 873(94.9) | 6 951(92.4) | 492(83.7) | 339 676(94.4) | 0.020 | 0.083 | 0.350 |
| Household income |  |  |  |  | 0.232 | 0.335 | 0.391 |
| <18 000, n(%) | 16 497(15.8) | 2 741(36.4) | 227(38.6) | 82 983(23.1) |  |  |  |
| 18 000-30 999, n(%) | 23 945(23.0) | 2 003(26.6) | 154(26.2) | 94 121(26.2) |  |  |  |
| 31 000-51 999, n(%) | 30 165(28.9) | 1 567(20.8) | 122(20.7) | 92 659(25.8) |  |  |  |
| 52 000-100 000, n(%) | 26 266(25.2) | 961(12.8) | 70(11.9) | 70 470(19.6) |  |  |  |
| >100 000, n(%) | 7 328(7.0) | 251(3.3) | 15(2.6) | 19 438(5.4) |  |  |  |
| Deprivation index, mean(SD) | -1.46(2.95) | -0.43(3.40) | 0.16(3.54) | -1.32(3.09) | 0.049 | 0.273 | 0.443 |
| BMI, mean(SD), kg/m^2^ | 27.33(4.72) | 29.23(5.63) | 30.61(5.78) | 27.36(4.74) | 0.006 | 0.360 | 0.615 |
| Alcohol consumption |  |  |  |  | 0.124 | 0.209 | 0.316 |
| Daily or almost daily, n(%) | 20 416(19.6) | 1 414(18.8) | 96(16.3) | 73 209(20.4) |  |  |  |
| Three or four times a week, n(%) | 26 582(25.5) | 1353(18.0) | 92(15.6) | 81 886(22.8) |  |  |  |
| Once or twice a week, n(%) | 28 762(27.6) | 1 825(24.3) | 147(25.0) | 91 957(25.6) |  |  |  |
| One to three times a month, n(%) | 11 927(11.4) | 799(10.6) | 64(10.9) | 40 314(11.2) |  |  |  |
| Special occasions only or never, n(%) | 10 034(9.6) | 1 168(15.5) | 96(16.3) | 42 710(11.9) |  |  |  |
| Never, n(%) | 6 480(6.2) | 964(12.8) | 93(15.8) | 29 595(8.2) |  |  |  |
| Smoking status |  |  |  |  | 0.070 | 0.228 | 0.263 |
| Never smoker, n(%) | 59 921(57.5) | 3 335(44.3) | 250(42.5) | 199 721(55.5) |  |  |  |
| Previous smoker, n(%) | 35 500(34.1) | 3 115(41.4) | 260(44.2) | 122 502(34.1) |  |  |  |
| Current smoker, n(%) | 8 780(8.4) | 1 073(14.3) | 78(13.3) | 37 448(10.4) |  |  |  |
| Physical activity, mean(SD), MET minutes/week | 2529.22(2585.74) | 2623.26(2813.78) | 2707.49(2935.05) | 2686.42(2739.02) | 0.059 | 0.023 | 0.007 |
| Comorbidities |  |  |  |  |  |  |  |
| Hypertension, n(%) | 33 161(31.8) | 4 523(60.1) | 339(57.7) | 132 105(36.7) | 0.103 | 0.481 | 0.429 |
| Diabetes, n(%) | 6 579(6.3) | 1 678(22.3) | 127(21.6) | 27 531(7.7) | 0.053 | 0.419 | 0.403 |
| Renal failure, n(%) | 3 515(3.4) | 1 050(14.0) | 81(13.8) | 15 075(4.2) | 0.043 | 0.345 | 0.340 |
| Myocardial infarction, n(%) | 3 599(3.5) | 890(11.8) | 68(11.6) | 15 720(4.4) | 0.047 | 0.276 | 0.268 |
| Stroke, n(%) | 2 056(2.0) | 623(8.3) | 27(4.6) | 9 577(2.7) | 0.046 | 0.249 | 0.103 |
| COPD, n(%) | 3 174(3.0) | 1 123(14.9) | 80(13.6) | 48 079(13.4) | 0.056 | 0.376 | 0.340 |
| Asthma, n(%) | 15 561(14.9) | 1 534(20.4) | 136(23.1) | 14 699(4.1) | 0.045 | 0.188 | 0.255 |
| Heart failure, n(%) | 1 607(1.5) | 714(9.5) | 35(6.0) | 7 888(2.2) | 0.048 | 0.315 | 0.191 |
| Dementia, n(%) | 732(0.7) | 321(4.3) | 7(1.2) | 2 322(0.6) | 0.007 | 0.236 | 0.057 |
| Recent hospital admissions, mean(SD) | 0.8(1.5) | 3.5(4.0) | 2.1(2.8) | 0.9(1.7) | 0.036 | 0.854 | 0.543 |
| History of previous digestive diseases, n(%) | 33 024(31.7) | 4 085(54.3) | 266(45.2) | 116 937(32.5) | 0.018 | 0.451 | 0.263 |

SMD: standard mean difference; BMI: body mass index; MET: metabolic equivalent of task; COPD: chronic obstructive pulmonary disease; SD: standard deviation

Table S7. Baseline characteristics of COVID-19, contemporary comparisons by severity of COVID-19 after weighting

| Characteristics | Non-hospitalized COVID  (n= 104 201) | Hospitalized COVID  (n= 7 523) | Severe COVID  (n= 588) | Contemporary comparisons  (n = 359 671) | SMD | | |
| --- | --- | --- | --- | --- | --- | --- | --- |
|  |  |  |  |  | Non-hospitalized COVID and contemporary comparisons | Hospitalized COVID and contemporary comparisons | Severe COVID and contemporary comparisons |
| Age, mean(SD), years | 56.1(8.0) | 56.9(8.3) | 57.5(7.7) | 56.2(8.1) | 0.003 | 0.004 | 0.091 |
| Sex, female, n(%) | 57415(55.1) | 4002(53.2) | 290(49.4) | 198 538(55.2) | 0.007 | 0.040 | 0.120 |
| Ethnicity, White, n(%) | 98574(94.6) | 7072(94) | 550(93.5) | 339 889(94.5) | 0.001 | 0.019 | 0.039 |
| Household income |  |  |  |  | 0.005 | 0.029 | 0.090 |
| <18 000, n(%) | 22195(21.3) | 1836(24.4) | 148(25.2) | 78 049(21.7) |  |  |  |
| 18 000-30 999, n(%) | 26467(25.4) | 1941(25.8) | 167(28.4) | 91 716(25.5) |  |  |  |
| 31 000-51 999, n(%) | 27613(26.5) | 1948(25.9) | 143(24.4) | 94 953(26.4) |  |  |  |
| 52 000-100 000, n(%) | 21882(21) | 1399(18.6) | 102(17.3) | 74 452(20.7) |  |  |  |
| >100 000, n(%) | 6044(5.8) | 391(5.2) | 28(4.8) | 20 501(5.7) |  |  |  |
| Deprivation index, mean(SD) | -1.4(3.0) | -1.2(3.1) | -1.1(3.2) | -1.3(3.1) | 0.006 | 0.040 | 0.067 |
| BMI, mean(SD), kg/m^2^ | 27.3(4.7) | 27.8(5.0) | 28.3(4.7) | 27.4(4.8) | 0.003 | 0.088 | 0.206 |
| Alcohol consumption |  |  |  |  | 0.007 | 0.048 | 0.166 |
| Daily or almost daily, n(%) | 21257(20.4) | 1550(20.6) | 106(18.1) | 72 654(20.2) |  |  |  |
| Three or four times a week, n(%) | 24487(23.5) | 1625(21.6) | 118(20.1) | 83 803(23.3) |  |  |  |
| Once or twice a week, n(%) | 27092(26) | 1836(24.4) | 145(24.6) | 93 514(26) |  |  |  |
| One to three times a month, n(%) | 11671(11.2) | 895(11.9) | 82(14) | 40 283(11.2) |  |  |  |
| Special occasions only or never, n(%) | 11671(11.2) | 963(12.8) | 65(11) | 41 002(11.4) |  |  |  |
| Never, n(%) | 8128(7.8) | 662(8.8) | 71(12.1) | 28 414(7.9) |  |  |  |
| Smoking status |  |  |  |  | 0.004 | 0.041 | 0.071 |
| Never smoker, n(%) | 58353(56) | 4002(53.2) | 320(54.5) | 200 696(55.8) |  |  |  |
| Previous smoker, n(%) | 35533(34.1) | 2693(35.8) | 193(32.8) | 123 007(34.2) |  |  |  |
| Current smoker, n(%) | 10212(9.8) | 835(11.1) | 75(12.7) | 35 967(10) |  |  |  |
| Physical activity, mean(SD), MET minutes/week | 2644.4(2696.9) | 2640.2(2719.5) | 2651.6(2725.8) | 2649.9(2708.2) | 0.002 | 0.016 | 0.013 |
| Comorbidities |  |  |  |  |  |  |  |
| Hypertension, n(%) | 37096(35.6) | 2979(39.6) | 253(43.1) | 129 841(36.1) | <0.001 | 0.046 | 0.130 |
| Diabetes, n(%) | 7711(7.4) | 700(9.3) | 66(11.2) | 27 335(7.6) | <0.001 | 0.041 | 0.122 |
| Renal failure, n(%) | 4168(4) | 414(5.5) | 32(5.4) | 15 106(4.2) | <0.001 | 0.048 | 0.058 |
| Myocardial infarction, n(%) | 4376(4.2) | 406(5.4) | 36(6.2) | 15 466(4.3) | <0.001 | 0.038 | 0.083 |
| Stroke, n(%) | 2605(2.5) | 256(3.4) | 21(3.6) | 9 351(2.6) | 0.002 | 0.033 | 0.051 |
| COPD, n(%) | 4064(3.9) | 429(5.7) | 36(6.2) | 14 747(4.1) | 0.002 | 0.057 | 0.095 |
| Asthma, n(%) | 14380(13.8) | 1106(14.7) | 98(16.6) | 49 635(13.8) | 0.001 | 0.032 | 0.089 |
| Heart failure, n(%) | 2188(2.1) | 263(3.5) | 28(4.7) | 7 913(2.2) | 0.002 | 0.060 | 0.135 |
| Dementia, n(%) | 729(0.7) | 90(1.2) | 2(0.4) | 2 518(0.7) | 0.004 | 0.042 | 0.040 |
| Recent hospital admissions, mean(SD) | 0.9(1.6) | 1.4(1.9) | 1.3(1.9) | 0.9(1.8) | 0.003 | 0.209 | 0.260 |
| History of previous digestive diseases, n(%) | 33761(32.4) | 2731(36.3) | 231(39.3) | 117 612(32.7) | 0.001 | 0.067 | 0.141 |

SMD: standard mean difference; BMI: body mass index; MET: metabolic equivalent of task; COPD: chronic obstructive pulmonary disease; SD: standard deviation

Table S8. Baseline characteristics of COVID-19 group and contemporary comparisons by status of SARS-CoV reinfection before weighting.

| Characteristics | Single SARS-CoV-2 infection group  (n= 107 950) | Reinfected  group  (n= 4 361) | Non-infected comparisons  (n = 359 671) | SMD | |
| --- | --- | --- | --- | --- | --- |
|  |  |  |  | Single SARS-CoV-2 infection group and Non-infected comparisons | Reinfected group and Non-infected comparisons |
| Age, mean(SD), years | 54.55(8.17) | 51.34(8.11) | 56.79(7.97) | 0.278 | 0.679 |
| Sex, female, n(%) | 58 918(54.6) | 2 577(59.1) | 199 368(55.4) | 0.017 | 0.074 |
| Ethnicity, White, n(%) | 102 257(94.7) | 4 058(93.1) | 339 676(94.4) | 0.013 | 0.057 |
| Household income |  |  |  | 0.189 | 0.209 |
| <18 000, n(%) | 18 695(17.3) | 770(17.7) | 82 983(23.1) |  |  |
| 18 000-30 999, n(%) | 25 173(23.3) | 929(21.3) | 94 121(26.2) |  |  |
| 31 000-51 999, n(%) | 30 510(28.3) | 1 343(30.8) | 92 659(25.8) |  |  |
| 52 000-100 000, n(%) | 26 261(24.3) | 1 036(23.8) | 70 470(19.6) |  |  |
| >100 000, n(%) | 7 311(6.8) | 283(6.5) | 19 438(5.4) |  |  |
| Deprivation index, mean(SD) | -1.40(2.99) | -0.95(3.14) | -1.32(3.09) | 0.028 | 0.117 |
| BMI, mean(SD), kg/m^2^ | 27.47(4.81) | 27.64(5.15) | 27.36(4.74) | 0.023 | 0.058 |
| Alcohol consumption |  |  |  | 0.100 | 0.132 |
| Daily or almost daily, n(%) | 21 191(19.6) | 734(16.8) | 73 209(20.4) |  |  |
| Three or four times a week, n(%) | 27 044(25.1) | 983(22.5) | 81 886(22.8) |  |  |
| Once or twice a week, n(%) | 29 470(27.3) | 1 264(29.0) | 91 957(25.6) |  |  |
| One to three times a month, n(%) | 12 211(11.3) | 579(13.3) | 40 314(11.2) |  |  |
| Special occasions only or never, n(%) | 10 793(10.0) | 505(11.6) | 42 710(11.9) |  |  |
| Never, n(%) | 7 241(6.7) | 296(6.8) | 29 595(8.2) |  |  |
| Smoking status |  |  |  | 0.056 | 0.011 |
| Never smoker, n(%) | 61 076(56.6) | 2 429(55.7) | 199 721(55.5) |  |  |
| Previous smoker, n(%) | 37 408(34.7) | 1 467(33.6) | 122 502(34.1) |  |  |
| Current smoker, n(%) | 9 466(8.8) | 465(10.7) | 37 448(10.4) |  |  |
| Physical activity, mean(SD), MET minutes/week | 2533.87(2598.45) | 2600.62(2729.75) | 2686.42(2739.02) | 0.057 | 0.031 |
| Comorbidities |  |  |  |  |  |
| Hypertension, n(%) | 36 683(34.0) | 1 340(30.7) | 132 105(36.7) | 0.058 | 0.127 |
| Diabetes, n(%) | 8 026(7.4) | 358(8.2) | 27 531(7.7) | 0.008 | 0.021 |
| Renal failure, n(%) | 4 474(4.1) | 172(3.9) | 15 075(4.2) | 0.002 | 0.013 |
| Myocardial infarction, n(%) | 4 380(4.1) | 177(4.1) | 15 720(4.4) | 0.016 | 0.016 |
| Stroke, n(%) | 2 605(2.5) | 256(3.4) | 9 577(2.7) | 0.002 | 0.033 |
| COPD, n(%) | 4 064(3.9) | 429(5.7) | 48 079(13.4) | 0.002 | 0.057 |
| Asthma, n(%) | 14 380(13.8) | 1 106(14.7) | 14 699(4.1) | 0.001 | 0.032 |
| Heart failure, n(%) | 2 188(2.1) | 263(3.5) | 7 888(2.2) | 0.002 | 0.060 |
| Dementia, n(%) | 957(0.9) | 103(2.4) | 2 322(0.6) | 0.028 | 0.141 |
| Recent hospital admissions, mean(SD) | 1.00(1.92) | 1.16(2.33) | 0.9(1.7) | 0.073 | 0.145 |
| History of previous digestive diseases, n(%) | 35 880(33.2) | 1 495(34.3) | 116 937(32.5) | 0.015 | 0.038 |

SMD: standard mean difference; BMI: body mass index; MET: metabolic equivalent of task; COPD: chronic obstructive pulmonary disease; SD: standard deviation

Table S9. Baseline characteristics of COVID-19 group and contemporary comparisons by severity of SARS-CoV reinfection after weighting.

| Characteristics | Single SARS-CoV-2 infection group  (n= 107 950) | Reinfected  group  (n= 4 361) | Non-infected comparisons  (n = 359 671) | SMD | |
| --- | --- | --- | --- | --- | --- |
|  |  |  |  | Single SARS-CoV-2 infection group and Non-infected comparisons | Reinfected group and Non-infected comparisons |
| Age, mean(SD), years | 56.3(8.1) | 57.0(8.3) | 56.2(8.1) | 0.002 | 0.037 |
| Sex, female, n(%) | 59265(54.9) | 2 189(50.2) | 198 538(55.2) | 0.007 | 0.105 |
| Ethnicity, White, n(%) | 102 013(94.5) | 4 108(94.2) | 339 889(94.5) | 0.001 | 0.010 |
| Household income |  |  |  | 0.004 | 0.035 |
| <18 000, n(%) | 23 317(21.6) | 1 038(23.8) | 78 049(21.7) |  |  |
| 18 000-30 999, n(%) | 27 527(25.5) | 1 090(25) | 91 716(25.5) |  |  |
| 31 000-51 999, n(%) | 28 391(26.3) | 1 116(25.6) | 94 953(26.4) |  |  |
| 52 000-100 000, n(%) | 22 454(20.8) | 859(19.7) | 74 452(20.7) |  |  |
| >100 000, n(%) | 6 261(5.8) | 257(5.9) | 20 501(5.7) |  |  |
| Deprivation index, mean(SD) | -1.3(3.0) | -1.3(3.1) | -1.3(3.1) | 0.002 | 0.018 |
| BMI, mean(SD), kg/m^2^ | 27.4(4.7) | 27.4(4.7) | 27.4(4.8) | 0.001 | 0.010 |
| Alcohol consumption |  |  |  | 0.005 | 0.062 |
| Daily or almost daily, n(%) | 21 914(20.3) | 938(21.5) | 72 654(20.2) |  |  |
| Three or four times a week, n(%) | 25 260(23.4) | 986(22.6) | 83 803(23.3) |  |  |
| Once or twice a week, n(%) | 27 959(25.9) | 1 099(25.2) | 93 514(26) |  |  |
| One to three times a month, n(%) | 12 090(11.2) | 423(9.7) | 40 283(11.2) |  |  |
| Special occasions only or never, n(%) | 12 198(11.3) | 515(11.8) | 41 002(11.4) |  |  |
| Never, n(%) | 8 528(7.9) | 397(9.1) | 28 414(7.9) |  |  |
| Smoking status |  |  |  | 0.002 | 0.026 |
| Never smoker, n(%) | 60 128(55.7) | 2 412(55.3) | 200 696(55.8) |  |  |
| Previous smoker, n(%) | 37 027(34.3) | 1 461(33.5) | 123 007(34.2) |  |  |
| Current smoker, n(%) | 10 795(10) | 488(11.2) | 35 967(10) |  |  |
| Physical activity, mean(SD), MET minutes/week | 2644.6(2699.9) | 2693.4(2830.0) | 2649.9(2708.2) | 0.002 | 0.003 |
| Comorbidities |  |  |  |  |  |
| Hypertension, n(%) | 38 970(36.1) | 1 683(38.6) | 129 841(36.1) | 0.001 | 0.039 |
| Diabetes, n(%) | 8 204(7.6) | 371(8.5) | 27 335(7.6) | 0.001 | 0.032 |
| Renal failure, n(%) | 4 534(4.2) | 170(3.9) | 15 106(4.2) | 0.001 | 0.013 |
| Myocardial infarction, n(%) | 4 642(4.3) | 214(4.9) | 15 466(4.3) | 0.001 | 0.026 |
| Stroke, n(%) | 2 807(2.6) | 131(3) | 9 351(2.6) | 0.002 | 0.022 |
| COPD, n(%) | 4 426(4.1) | 218(5) | 14 747(4.1) | 0.003 | 0.043 |
| Asthma, n(%) | 15 005(13.9) | 593(13.6) | 49 635(13.8) | 0.002 | 0.004 |
| Heart failure, n(%) | 2 375(2.2) | 100(2.3) | 7 913(2.2) | 0.002 | 0.007 |
| Dementia, n(%) | 756(0.7) | 35(0.8) | 2 518(0.7) | 0.002 | 0.017 |
| Recent hospital admissions, mean(SD) | 0.9(1.7) | 1.0(1.8) | 0.9(1.8) | 0.009 | 0.081 |
| History of previous digestive diseases, n(%) | 72 542(67.2) | 2 913(66.8) | 117 612(32.7) | 0.003 | 0.014 |

SMD: standard mean difference; BMI: body mass index; MET: metabolic equivalent of task; COPD: chronic obstructive pulmonary disease; SD: standard deviation

Table S10. Hazard ratio of digestive outcomes in the reinfected group, single SARS-CoV-2 infection group, and non-infected comparisons.

| Outcome | Single SARS-CoV-2 infection group vs Non-infected comparisons | | Reinfected group vs Non-infected comparisons | |
| --- | --- | --- | --- | --- |
|  | HR (95% CI) | P value | HR (95% CI) | P value |
| GI dysfunction | 1.44(1.29,1.62) | **<0.001** | 0.80(0.43,1.47) | 0.469 |
| PUD | 1.65(1.28,2.13) | **<0.001** | 0.73(0.22,2.39) | 0.598 |
| GERD | 1.44(1.29,1.60) | **<0.001** | 1.41(0.72,2.75) | 0.317 |
| IBD | 1.65(1.09,2.51) | **0.018** | 2.85(0.59,13.82) | 0.194 |
| Severe liver disease | 1.28(1.08,1.53) | **0.005** | 0.60(0.22,1.62) | 0.316 |
| NAFLD | 1.12(0.77,1.61) | 0.553 | 3.34(0.9,12.46) | 0.072 |
| Gallbladder disease | 1.19(0.98,1.44) | 0.081 | 1.22(0.39,3.81) | 0.737 |
| Pancreatic disease | 1.44(1.11,1.88) | **0.007** | 5.40(2.22,13.15) | **<0.001** |

GI, gastrointestinal;GERD, gastroesophageal reflux disease; PUD, peptic ulcer disease; IBD, inflammatory bowel disease; NAFLD, non-alcohol fatty liver disease;

HR: hazard ratio; CI: confidence interval;

Outcomes were ascertained 30 days after the COVID-19-positive test until the end of follow-up. Weighted HRs after IPTW and 95% CIs are presented.

Table S11. Hazard ratio of digestive outcomes in reinfected group and single SARS-CoV-2 infection group in head-to-head comparison.

| Outcome | Reinfected group vs Single SARS-CoV-2 infection group | |
| --- | --- | --- |
|  | HR (95% CI) | P value |
| GI dysfunction | 1.12(0.65,1.91) | 0.685 |
| PUD | 0.95(0.30,3.01) | 0.928 |
| GERD | 1.13(0.72,1.77) | 0.606 |
| IBD | 1.77(0.42,7.47) | 0.436 |
| Severe liver disease | 1.90(0.58,6.24) | 0.293 |
| NAFLD | 0.90(0.37,2.20) | 0.812 |
| Gallbladder disease | 0.94(0.42,2.14) | 0.890 |
| Pancreatic disease | 2.57(1.23,5.38) | 0.012 |

HR: hazard ratio; CI: confidence interval;

Outcomes were ascertained 30 days after the COVID-19-positive test until the end of follow-up. Weighted HRs after IPTW and 95% CIs are presented.

Table S12. Baseline characteristics of COVID-19 group and contemporary comparisons in the sensitive analysis restricting to the period before vaccination was available before weighting.

| Characteristics | COVID-19 group  (n = 8 431) | Contemporary comparisons  (n = 359 671) | SMD |
| --- | --- | --- | --- |
| Age, mean(SD), years | 54.39(8.82) | 56.79(7.97) | 0.286 |
| Sex, female, n(%) | 4 280(50.8) | 199 368(55.4) | 0.094 |
| Ethnicity, White, n(%) | 7 771(92.2) | 339 676(94.4) | 0.091 |
| Household income |  |  | 0.073 |
| <18 000, n(%) | 2 147(25.5) | 82 983(23.1) |  |
| 18 000-30 999, n(%) | 2 119(25.1) | 94 121(26.2) |  |
| 31 000-51 999, n(%) | 2 236(26.5) | 92 659(25.8) |  |
| 52 000-100 000, n(%) | 1 546(18.3) | 70 470(19.6) |  |
| >100 000, n(%) | 383(4.5) | 19 438(5.4) |  |
| Deprivation index, mean(SD) | -0.73(3.27) | -1.32(3.09) | 0.184 |
| BMI, mean(SD), kg/m^2^ | 28.43(5.07) | 27.36(4.74) | 0.218 |
| Alcohol consumption |  |  | 0.114 |
| Daily or almost daily, n(%) | 1 408(16.7) | 73 209(20.4) |  |
| Three or four times a week, n(%) | 1 843(21.9) | 81 886(22.8) |  |
| Once or twice a week, n(%) | 2 394(28.4) | 91 957(25.6) |  |
| One to three times a month, n(%) | 965(11.4) | 40 314(11.2) |  |
| Special occasions only or never, n(%) | 1 004(11.9) | 42 710(11.9) |  |
| Never, n(%) | 817(9.7) | 29 595(8.2) |  |
| Smoking status |  |  | 0.086 |
| Never smoker, n(%) | 4 322(51.3) | 199 721(55.5) |  |
| Previous smoker, n(%) | 3 115(36.9) | 122 502(34.1) |  |
| Current smoker, n(%) | 994(11.8) | 37 448(10.4) |  |
| Physical activity, mean(SD), MET minutes/week | 2734.76(2828.4) | 2686.42(2739.0) | 0.017 |
| Comorbidities |  |  |  |
| Hypertension, n(%) | 3 411(40.5) | 132 105(36.7) | 0.077 |
| Diabetes, n(%) | 1 005(11.9) | 27 531(7.7) | 0.144 |
| Renal failure, n(%) | 573(6.8) | 15 075(4.2) | 0.115 |
| Myocardial infarction, n(%) | 547(6.5) | 15 720(4.4) | 0.094 |
| Stroke, n(%) | 398(4.7) | 9 577(2.7) | 0.109 |
| COPD, n(%) | 616(7.3) | 14 699(4.1) | 0.139 |
| Asthma, n(%) | 1 382(16.4) | 48 079(13.4) | 0.085 |
| Heart failure, n(%) | 385(4.6) | 7 888(2.2) | 0.132 |
| Dementia, n(%) | 345(4.1) | 2 322(0.6) | 0.228 |
| Recent hospital admissions, mean(SD) | 1.6(2.9) | 0.9(1.7) | 0.315 |
| History of previous digestive diseases, n(%) | 3 286(39.0) | 116 937(32.5) | 0.135 |

SMD: standard mean difference; BMI: body mass index; MET: metabolic equivalent of task; COPD: chronic obstructive pulmonary disease; SD: standard deviation

Table S13. Baseline characteristics of COVID-19 group and contemporary comparisons in the sensitive analysis restricting to the period before vaccination was available after weighting.

| Characteristics | COVID-19 group  (n = 8 431) | Contemporary comparisons  (n = 359 671) | SMD |
| --- | --- | --- | --- |
| Age, mean(SD), years | 56.8(8.5) | 56.9(8.0) | 0.011 |
| Sex, female, n(%) | 4 494(53.3) | 197 459(54.9) | 0.031 |
| Ethnicity, White, n(%) | 7 942(94.2) | 339 529(94.4) | 0.010 |
| Household income |  |  | 0.024 |
| <18 000, n(%) | 2 066(24.5) | 84 882(23.6) |  |
| 18 000-30 999, n(%) | 2 175(25.8) | 94 234(26.2) |  |
| 31 000-51 999, n(%) | 2 133(25.3) | 92 076(25.6) |  |
| 52 000-100 000, n(%) | 1 593(18.9) | 69 417(19.3) |  |
| >100 000, n(%) | 464(5.5) | 19 063(5.3) |  |
| Deprivation index, mean(SD) | -1.2(3.1) | -1.3(3.1) | 0.037 |
| BMI, mean(SD), kg/m^2^ | 27.6(4.6) | 27.4(4.8) | 0.047 |
| Alcohol consumption |  |  | 0.014 |
| Daily or almost daily, n(%) | 1 728(20.5) | 73 373(20.4) |  |
| Three or four times a week, n(%) | 1 905(22.6) | 81 286(22.6) |  |
| Once or twice a week, n(%) | 2 125(25.2) | 91 716(25.5) |  |
| One to three times a month, n(%) | 936(11.1) | 40 283(11.2) |  |
| Special occasions only or never, n(%) | 1 012(12.0) | 42 801(11.9) |  |
| Never, n(%) | 733(8.7) | 30 212(8.4) |  |
| Smoking status |  |  | 0.034 |
| Never smoker, n(%) | 4 519(53.6) | 197 459(54.9) |  |
| Previous smoker, n(%) | 2 926(34.7) | 123 727(34.4) |  |
| Current smoker, n(%) | 978(11.6) | 38 485(10.7) |  |
| Physical activity, mean(SD), MET minutes/week | 2682.4(2718.9) | 2684.3(2744.4) | 0.001 |
| Comorbidities |  |  |  |
| Hypertension, n(%) | 3 280(38.9) | 135 236(37.6) | 0.027 |
| Diabetes, n(%) | 742(8.8) | 29 493(8.2) | 0.023 |
| Renal failure, n(%) | 396(4.7) | 16 905(4.7) | 0.004 |
| Myocardial infarction, n(%) | 405(4.8) | 17 264(4.8) | 0.001 |
| Stroke, n(%) | 295(3.5) | 11 150(3.1) | 0.022 |
| COPD, n(%) | 438(5.2) | 16 905(4.7) | 0.021 |
| Asthma, n(%) | 1 172(13.9) | 48 915(13.6) | 0.010 |
| Heart failure, n(%) | 253(3.0) | 10 071(2.8) | 0.013 |
| Dementia, n(%) | 101(1.2) | 3 956(1.1) | 0.012 |
| Recent hospital admissions, mean(SD) | 1.0(1.7) | 0.9(1.7) | 0.051 |
| History of previous digestive diseases, n(%) | 2 496(29.6) | 107 182(29.8) | 0.005 |

SMD: standard mean difference; BMI: body mass index; MET: metabolic equivalent of task; COPD: chronic obstructive pulmonary disease; SD: standard deviation

Table S14. Hazard ratio of digestive outcomes in COVID-19 group and contemporary and historical comparisons in subgroups in the sensitive analysis restricting to the period before vaccination was available.

| Outcome | COVID-19 vs Contemporary comparison | | COVID-19 vs Historical comparison | |
| --- | --- | --- | --- | --- |
|  | HR (95% CI) | P value | HR (95% CI) | P value |
| GI dysfunction | 3.92(2.33,6.60) | **<0.001** | 3.47(2.06,5.84) | **<0.001** |
| PUD | 2.45(0.56,10.67) | 0.233 | 1.82(0.40,8.23) | 0.437 |
| GERD | 1.26(0.50,3.16) | 0.625 | 1.03(0.41,2.63) | 0.944 |
| IBD | 6.27(1.20,32.86) | **0.030** | 4.00(0.78,20.6) | 0.097 |
| Severe liver disease | 2.51(1.30,4.85) | **0.006** | 2.53(1.30,4.92) | **0.006** |
| NAFLD | 1.49(0.29,7.60) | 0.630 | 1.59(0.30,8.39) | 0.586 |
| Gallbladder disease | 2.86(1.02,8.07) | **0.047** | 3.35(1.15,9.72) | **0.026** |
| Pancreatic disease | 3.52(1.21,10.25) | **0.021** | 3.58(1.20,10.71) | **0.023** |

GI, gastrointestinal;GERD, gastroesophageal reflux disease; PUD, peptic ulcer disease; IBD, inflammatory bowel disease; NAFLD, non-alcohol fatty liver disease;

HR: hazard ratio; CI: confidence interval;

Outcomes were ascertained 30 days after the COVID-19-positive test until the end of follow-up. Weighted HRs after IPTW and 95% CIs are presented.

Table S15. Hazard ratio of digestive outcomes in COVID-19 group compared to the contemporary and historical comparisons by pooling estimates across all five imputed datasets.

| **Outcome** | **COVID-19 group vs contemporary comparison (REF)** | | **COVID-19 group vs historical comparison (REF)** | |
| --- | --- | --- | --- | --- |
|  | **HR (95% CI)** | **P value** | **HR (95% CI)** | **P value** |
| **GI dysfunction** | 1.38(1.26,1.50) | <0.001 | 1.20(1.10,1.31) | <0.001 |
| Dyspepsia | 1.50(1.01,2.05) | 0.010 | 1.03(0.77,1.39) | 0.826 |
| IBS | 1.33(1.10,1.62) | 0.004 | 1.07(0.88,1.29) | 0.508 |
| Constipation | 1.41(1.29,1.55) | <0.001 | 1.27(1.16,1.39) | <0.001 |
| **PUD** | 1.23(1.00,1.51) | 0.049 | 0.96(0.78,1.17) | 0.667 |
| Gastric ulcer | 1.43(1.14,1.81) | 0.002 | 1.05(0.84,1.32) | 0.669 |
| Duodenal ulcer | 0.97(0.67,1.39) | 0.868 | 0.80(0.56,1.15) | 0.222 |
| Other PUD | 0.85(0.35,2.06) | 0.702 | 0.60(0.26,1.41) | 0.235 |
| **GERD** | 1.41(1.30,1.53) | <0.001 | 1.22(1.13,1.32) | <0.001 |
| **IBD** | 1.35(0.99,1.84) | 0.062 | 1.20(0.88,1.63) | 0.253 |
| CD | 1.31(0.83,2.07) | 0.238 | 1.14(0.73,1.79) | 0.557 |
| UC | 1.15(0.78,1.70) | 0.476 | 1.01(0.69,1.49) | 0.944 |
| **Gallbladder disease** | 1.21(1.06,1.38) | 0.004 | 1.12(0.99,1.28) | 0.079 |
| Cholelithiasis | 1.21(1.06,1.39) | 0.005 | 1.12(0.98,1.28) | 0.110 |
| Cholecystitis | 1.60(1.21,2.12) | 0.001 | 1.49(1.13,1.97) | 0.005 |
| **Severe liver disease** | 1.35(1.03,1.77) | 0.029 | 1.18(0.90,1.54) | 0.234 |
| **NAFLD** | 1.27(1.10,1.48) | 0.001 | 1.66(1.42,1.93) | <0.001 |
| **Pancreatic disease** | 1.36(1.11,1.66) | 0.003 | 1.48(1.20,1.81) | <0.001 |
| Acute pancreatitis | 1.49(1.12,1.98) | 0.007 | 1.64(1.22,2.20) | 0.001 |
| Chronic pancreatitis | 1.13(0.66,1.94) | 0.649 | 0.95(0.56,1.64) | 0.864 |
| Pancreatic cyst | 1.49(0.97,2.29) | 0.066 | 1.55(1.00,2.41) | 0.048 |
| Other pancreatic diseases | 1.07(0.76,1.49) | 0.697 | 1.27(0.90,1.79) | 0.172 |

GI, gastrointestinal; IBS, irritable bowel syndrome; PUD, peptic ulcer disease; IBD, inflammatory bowel disease; CD, Crohn’s disease; UC, ulcerative colitis; NAFLD, non-alcohol fatty liver disease;

HR: hazard ratios; CI: confidence interval; Outcomes were ascertained 30 days after the COVID-19-positive test until the end of follow-up. Weighted HRs after IPTW and 95% CIs are presented.

Table S16. Hazard ratio of digestive outcomes compared with contemporary and historical comparisons in subgroups

| **Outcome** | **Subgroup** | **COVID-19 vs Contemporary comparison** | | | **COVID-19 vs Historical comparison** | | |
| --- | --- | --- | --- | --- | --- | --- | --- |
|  |  | **HR** | **95% CI** | **Pi** | **HR** | **95% CI** | **Pi** |
|  | **Age** |  |  |  |  |  |  |
| **GI dysfunction** | Age<60 | 1.37 | 1.20,1.56 | 0.555 | 1.14 | 1.00,1.30 | 0.123 |
|  | Age>=60 | 1.44 | 1.28,1.62 |  | 1.30 | 1.16,1.47 |  |
| **PUD** | Age<60 | 1.21 | 0.91,1.61 | 0.757 | 1.00 | 0.75,1.32 | 0.858 |
|  | Age>=60 | 1.28 | 0.95,1.71 |  | 0.95 | 0.72,1.27 |  |
| **GERD** | Age<60 | 1.35 | 1.21,1.50 | 0.173 | 1.27 | 1.15,1.42 | 0.504 |
|  | Age>=60 | 1.52 | 1.34,1.72 |  | 1.21 | 1.07,1.37 |  |
| **IBD** | Age<60 | 1.04 | 0.69,1.57 | 0.070 | 1.10 | 0.72,1.68 | 0.540 |
|  | Age>=60 | 1.84 | 1.16,2.91 |  | 1.33 | 0.86,2.07 |  |
| **Severe liver disease** | Age<60 | 1.24 | 0.85,1.79 | 0.487 | 1.06 | 0.73,1.53 | 0.426 |
|  | Age>=60 | 1.49 | 1.01,2.20 |  | 1.32 | 0.90,1.93 |  |
| **NAFLD** | Age<60 | 1.16 | 0.96,1.40 | 0.146 | 1.46 | 1.20,1.79 | 0.082 |
|  | Age>=60 | 1.44 | 1.14,1.81 |  | 1.94 | 1.52,2.46 |  |
| **Gallbladder disease** | Age<60 | 1.19 | 1.00,1.41 | 0.630 | 1.05 | 0.89,1.25 | 0.239 |
|  | Age>=60 | 1.27 | 1.05,1.54 |  | 1.24 | 1.02,1.50 |  |
| **Pancreatic disease** | Age<60 | 1.42 | 1.07,1.88 | 0.805 | 1.51 | 1.13,2.02 | 0.970 |
|  | Age>=60 | 1.37 | 1.03,1.82 |  | 1.52 | 1.14,2.03 |  |
|  | **Ethnicity** |  |  |  |  |  |  |
| **GI dysfunction** | White | 1.37 | 1.25,1.51 | 0.754 | 1.21 | 1.10,1.32 | 0.891 |
|  | Other | 1.48 | 1.02,2.16 |  | 1.19 | 0.83,1.72 |  |
| **PUD** | White | 1.21 | 0.98,1.50 | 0.563 | 0.95 | 0.77,1.17 | 0.715 |
|  | Other | 1.44 | 0.67,3.10 |  | 1.04 | 0.49,2.17 |  |
| **GERD** | White | 1.43 | 1.31,1.56 | 0.257 | 1.23 | 1.14,1.34 | 0.397 |
|  | Other | 1.16 | 0.83,1.62 |  | 1.06 | 0.76,1.47 |  |
| **IBD** | White | 1.41 | 1.03,1.94 | 0.155 | 1.29 | 0.94,1.76 | 0.050 |
|  | Other | 0.43 | 0.09,2.12 |  | 0.28 | 0.06,1.29 |  |
| **Severe liver disease** | White | 1.40 | 1.07,1.84 | 0.336 | 1.21 | 0.93,1.59 | 0.403 |
|  | Other | 0.72 | 0.16,3.17 |  | 0.68 | 0.16,2.96 |  |
| **NAFLD** | White | 1.24 | 1.07,1.45 | 0.516 | 1.62 | 1.38,1.91 | 0.664 |
|  | Other | 1.47 | 0.87,2.51 |  | 1.79 | 1.03,3.11 |  |
| **Gallbladder disease** | White | 1.20 | 1.05,1.37 | 0.453 | 1.12 | 0.98,1.28 | 0.853 |
|  | Other | 1.51 | 0.86,2.66 |  | 1.20 | 0.69,2.09 |  |
| **Pancreatic disease** | White | 1.35 | 1.10,1.66 | 0.649 | 1.50 | 1.21,1.85 | 0.688 |
|  | Other | 1.66 | 0.71,3.88 |  | 1.25 | 0.55,2.84 |  |
|  | **Smoking**  **status** |  |  |  |  |  |  |
| **GI dysfunction** | Never smoke | 1.29 | 1.14,1.47 |  | 1.18 | 1.04,1.34 |  |
|  | Previous smoke | 1.42 | 1.23,1.63 | 0.338 | 1.17 | 1.02,1.35 | 0.979 |
|  | Current smoke | 1.56 | 1.21,2.02 | 0.200 | 1.39 | 1.08,1.79 | 0.270 |
| **PUD** | Never smoke | 1.21 | 0.90,1.64 |  | 0.95 | 0.71,1.28 |  |
|  | Previous smoke | 1.33 | 0.97,1.82 | 0.674 | 1.06 | 0.78,1.44 | 0.624 |
|  | Current smoke | 1.02 | 0.55,1.90 | 0.599 | 0.72 | 0.39,1.32 | 0.401 |
| **GERD** | Never smoke | 1.37 | 1.23,1.54 |  | 1.26 | 1.13,1.41 |  |
|  | Previous smoke | 1.40 | 1.23,1.61 | 0.811 | 1.11 | 0.97,1.26 | 0.143 |
|  | Current smoke | 1.60 | 1.25,2.03 | 0.279 | 1.47 | 1.16,1.86 | 0.257 |
| **IBD** | Never smoke | 0.94 | 0.55,1.61 |  | 0.88 | 0.51,1.52 |  |
|  | Previous smoke | 1.69 | 1.08,2.64 | 0.103 | 1.37 | 0.89,2.12 | 0.217 |
|  | Current smoke | 1.53 | 0.73,3.20 | 0.293 | 1.47 | 0.70,3.12 | 0.268 |
| **Severe liver disease** | Never smoke | 1.23 | 0.80,1.91 |  | 0.96 | 0.62,1.47 |  |
|  | Previous smoke | 1.58 | 1.07,2.34 | 0.408 | 1.59 | 1.07,2.35 | 0.087 |
|  | Current smoke | 0.99 | 0.49,1.98 | 0.611 | 0.85 | 0.43,1.68 | 0.776 |
| **NAFLD** | Never smoke | 1.36 | 1.09,1.68 |  | 1.68 | 1.34,2.10 |  |
|  | Previous smoke | 1.18 | 0.93,1.49 | 0.410 | 1.53 | 1.20,1.96 | 0.622 |
|  | Current smoke | 1.23 | 0.83,1.84 | 0.630 | 1.97 | 1.28,3.04 | 0.561 |
| **Gallbladder disease** | Never smoke | 1.28 | 1.06,1.53 |  | 1.18 | 0.98,1.41 |  |
|  | Previous smoke | 1.17 | 0.95,1.45 | 0.587 | 1.07 | 0.87,1.32 | 0.540 |
|  | Current smoke | 1.07 | 0.73,1.57 | 0.418 | 1.07 | 0.72,1.59 | 0.694 |
| **Pancreatic disease** | Never smoke | 1.33 | 0.99,1.78 |  | 1.51 | 1.12,2.04 |  |
|  | Previous smoke | 1.49 | 1.08,2.06 | 0.646 | 1.50 | 1.08,2.08 | 0.948 |
|  | Current smoke | 1.11 | 0.64,1.90 | 0.532 | 1.31 | 0.76,2.26 | 0.627 |
|  | **Alcohol consumption** |  |  |  |  |  |  |
| **GI dysfunction** | Daily or almost daily | 1.29 | 1.05,1.57 |  | 1.16 | 0.95,1.41 |  |
|  | Three or four times a week | 1.33 | 1.10,1.61 | 0.781 | 1.24 | 1.02,1.50 | 0.560 |
|  | Once or twice a week | 1.40 | 1.17,1.68 | 0.500 | 1.14 | 0.96,1.36 | 0.954 |
|  | One to three times a month | 1.13 | 0.86,1.49 | 0.482 | 0.95 | 0.72,1.25 | 0.273 |
|  | Special occasions only | 1.44 | 1.12,1.85 | 0.470 | 1.29 | 1.01,1.65 | 0.468 |
|  | Never | 1.74 | 1.34,2.27 | 0.071 | 1.50 | 1.16,1.94 | 0.110 |
| **PUD** | Daily or almost daily | 1.21 | 0.77,1.89 |  | 1.03 | 0.66,1.59 |  |
|  | Three or four times a week | 1.26 | 0.81,1.95 | 0.964 | 0.96 | 0.63,1.46 | 0.764 |
|  | Once or twice a week | 1.07 | 0.70,1.65 | 0.619 | 0.89 | 0.58,1.36 | 0.601 |
|  | One to three times a month | 0.96 | 0.51,1.82 | 0.507 | 0.74 | 0.39,1.40 | 0.382 |
|  | Special occasions only | 0.84 | 0.45,1.59 | 0.309 | 0.62 | 0.33,1.14 | 0.166 |
|  | Never | 2.53 | 1.41,4.51 | 0.056 | 1.56 | 0.89,2.73 | 0.252 |
| **GERD** | Daily or almost daily | 1.30 | 1.07,1.59 |  | 1.06 | 0.87,1.29 |  |
|  | Three or four times a week | 1.36 | 1.14,1.61 | 0.751 | 1.39 | 1.17,1.66 | 0.038 |
|  | Once or twice a week | 1.49 | 1.28,1.75 | 0.276 | 1.25 | 1.08,1.46 | 0.166 |
|  | One to three times a month | 1.17 | 0.90,1.51 | 0.531 | 1.10 | 0.85,1.42 | 0.790 |
|  | Special occasions only | 1.39 | 1.11,1.74 | 0.645 | 1.30 | 1.04,1.62 | 0.170 |
|  | Never | 1.90 | 1.45,2.48 | 0.028 | 1.13 | 0.88,1.45 | 0.703 |
| **IBD** | Daily or almost daily | 1.32 | 0.62,2.81 |  | 1.04 | 0.49,2.18 |  |
|  | Three or four times a week | 1.18 | 0.57,2.45 | 0.856 | 0.91 | 0.45,1.86 | 0.859 |
|  | Once or twice a week | 1.65 | 0.92,2.95 | 0.638 | 1.77 | 0.98,3.21 | 0.261 |
|  | One to three times a month | 1.37 | 0.62,3.04 | 0.937 | 1.40 | 0.62,3.19 | 0.574 |
|  | Special occasions only | 0.90 | 0.37,2.20 | 0.543 | 1.18 | 0.47,2.98 | 0.788 |
|  | Never | 1.86 | 0.69,5.00 | 0.569 | 0.89 | 0.36,2.21 | 0.828 |
| **Severe liver disease** | Daily or almost daily | 0.82 | 0.45,1.49 |  | 0.81 | 0.44,1.48 |  |
|  | Three or four times a week | 1.12 | 0.62,2.02 | 0.454 | 1.04 | 0.58,1.88 | 0.553 |
|  | Once or twice a week | 0.83 | 0.42,1.64 | 0.968 | 0.60 | 0.31,1.18 | 0.544 |
|  | One to three times a month | 3.55 | 1.47,8.58 | 0.007 | 2.58 | 1.18,5.63 | 0.019 |
|  | Special occasions only | 2.70 | 1.44,5.04 | 0.007 | 2.64 | 1.42,4.88 | 0.007 |
|  | Never | 1.57 | 0.67,3.65 | 0.202 | 1.17 | 0.52,2.65 | 0.468 |
| **NAFLD** | Daily or almost daily | 1.19 | 0.84,1.67 |  | 1.63 | 1.14,2.33 |  |
|  | Three or four times a week | 1.11 | 0.79,1.56 | 0.794 | 1.76 | 1.21,2.55 | 0.716 |
|  | Once or twice a week | 1.30 | 0.96,1.75 | 0.723 | 1.63 | 1.18,2.24 | 0.988 |
|  | One to three times a month | 1.35 | 0.87,2.10 | 0.670 | 1.61 | 1.02,2.55 | 0.976 |
|  | Special occasions only | 1.43 | 1.00,2.04 | 0.494 | 1.75 | 1.20,2.53 | 0.796 |
|  | Never | 1.19 | 0.77,1.85 | 0.949 | 1.33 | 0.85,2.07 | 0.544 |
| **Gallbladder disease** | Daily or almost daily | 1.25 | 0.89,1.75 |  | 1.09 | 0.78,1.54 |  |
|  | Three or four times a week | 0.96 | 0.71,1.29 | 0.288 | 0.98 | 0.72,1.32 | 0.632 |
|  | Once or twice a week | 1.38 | 1.08,1.76 | 0.586 | 1.16 | 0.91,1.47 | 0.777 |
|  | One to three times a month | 1.08 | 0.73,1.60 | 0.628 | 0.85 | 0.57,1.25 | 0.335 |
|  | Special occasions only | 1.43 | 1.05,1.95 | 0.507 | 1.53 | 1.12,2.08 | 0.146 |
|  | Never | 1.04 | 0.69,1.56 | 0.522 | 1.06 | 0.70,1.61 | 0.947 |
| **Pancreatic disease** | Daily or almost daily | 1.25 | 0.74,2.08 |  | 1.35 | 0.80,2.28 |  |
|  | Three or four times a week | 0.81 | 0.48,1.35 | 0.280 | 1.11 | 0.65,1.89 | 0.665 |
|  | Once or twice a week | 1.43 | 0.96,2.15 | 0.567 | 1.28 | 0.86,1.91 | 0.985 |
|  | One to three times a month | 1.41 | 0.79,2.51 | 0.668 | 1.58 | 0.88,2.85 | 0.610 |
|  | Special occasions only | 1.90 | 1.18,3.08 | 0.192 | 2.12 | 1.30,3.46 | 0.177 |
|  | Never | 1.79 | 1.03,3.11 | 0.292 | 1.88 | 1.05,3.39 | 0.350 |
|  | **Hypertension** |  |  |  |  |  |  |
| **GI dysfunction** | No | 1.36 | 1.20,1.54 | 0.799 | 1.31 | 1.16,1.49 | 0.080 |
|  | Yes | 1.39 | 1.23,1.58 |  | 1.12 | 0.99,1.27 |  |
| **PUD** | No | 1.51 | 1.15,2.00 | 0.047 | 1.39 | 1.05,1.83 | 0.001 |
|  | Yes | 0.99 | 0.72,1.35 |  | 0.69 | 0.51,0.94 |  |
| **GERD** | No | 1.45 | 1.31,1.62 | 0.400 | 1.49 | 1.34,1.66 | <0.001 |
|  | Yes | 1.35 | 1.19,1.54 |  | 0.99 | 0.88,1.12 |  |
| **IBD** | No | 0.93 | 0.58,1.49 | 0.026 | 0.91 | 0.57,1.46 | 0.117 |
|  | Yes | 1.91 | 1.25,2.91 |  | 1.50 | 1.00,2.24 |  |
| **Severe liver disease** | No | 1.11 | 0.70,1.75 | 0.294 | 1.14 | 0.71,1.82 | 0.877 |
|  | Yes | 1.49 | 1.07,2.08 |  | 1.18 | 0.86,1.64 |  |
| **NAFLD** | No | 1.22 | 0.97,1.53 | 0.668 | 1.83 | 1.43,2.35 | 0.323 |
|  | Yes | 1.30 | 1.07,1.57 |  | 1.55 | 1.27,1.90 |  |
| **Gallbladder disease** | No | 1.26 | 1.06,1.51 | 0.462 | 1.21 | 1.01,1.45 | 0.233 |
|  | Yes | 1.15 | 0.96,1.39 |  | 1.04 | 0.86,1.25 |  |
| **Pancreatic disease** | No | 1.24 | 0.92,1.68 | 0.418 | 1.66 | 1.21,2.28 | 0.358 |
|  | Yes | 1.46 | 1.12,1.92 |  | 1.36 | 1.03,1.78 |  |
|  | **Heart Failure** |  |  |  |  |  |  |
| **GI dysfunction** | No | 1.36 | 1.24,1.49 | 0.158 | 1.21 | 1.11,1.33 | 0.481 |
|  | Yes | 1.82 | 1.24,2.67 |  | 1.08 | 0.76,1.52 |  |
| **PUD** | No | 1.26 | 1.02,1.55 | 0.348 | 1.04 | 0.84,1.28 | 0.027 |
|  | Yes | 0.73 | 0.24,2.26 |  | 0.31 | 0.11,0.90 |  |
| **GERD** | No | 1.40 | 1.29,1.53 | 0.430 | 1.24 | 1.15,1.35 | 0.052 |
|  | Yes | 1.74 | 1.06,2.84 |  | 0.80 | 0.52,1.25 |  |
| **IBD** | No | 1.38 | 1.00,1.89 | 0.555 | 1.26 | 0.92,1.72 | 0.293 |
|  | Yes | 0.85 | 0.16,4.41 |  | 0.57 | 0.13,2.60 |  |
| **Severe liver disease** | No | 1.23 | 0.91,1.65 | 0.177 | 1.12 | 0.83,1.50 | 0.655 |
|  | Yes | 2.04 | 1.06,3.93 |  | 1.32 | 0.73,2.40 |  |
| **NAFLD** | No | 1.19 | 1.02,1.39 | 0.010 | 1.60 | 1.36,1.89 | 0.526 |
|  | Yes | 2.53 | 1.48,4.34 |  | 1.91 | 1.17,3.13 |  |
| **Gallbladder disease** | No | 1.24 | 1.08,1.41 | 0.096 | 1.17 | 1.03,1.34 | 0.019 |
|  | Yes | 0.69 | 0.35,1.34 |  | 0.52 | 0.27,1.01 |  |
| **Pancreatic disease** | No | 1.37 | 1.11,1.68 | 0.618 | 1.56 | 1.26,1.93 | 0.089 |
|  | Yes | 1.10 | 0.50,2.44 |  | 0.79 | 0.38,1.66 |  |
|  | **Renal Failure** |  |  |  |  |  |  |
| **GI dysfunction** | No | 1.37 | 1.24,1.50 | 0.556 | 1.25 | 1.14,1.37 | 0.039 |
|  | Yes | 1.51 | 1.12,2.03 |  | 0.92 | 0.70,1.21 |  |
| **PUD** | No | 1.24 | 1.00,1.53 | 0.951 | 0.99 | 0.80,1.23 | 0.434 |
|  | Yes | 1.21 | 0.61,2.42 |  | 0.75 | 0.39,1.47 |  |
| **GERD** | No | 1.43 | 1.32,1.56 | 0.131 | 1.29 | 1.19,1.41 | <0.001 |
|  | Yes | 1.06 | 0.73,1.54 |  | 0.57 | 0.40,0.82 |  |
| **IBD** | No | 1.35 | 0.97,1.87 | 0.997 | 1.20 | 0.87,1.66 | 1.000 |
|  | Yes | 1.35 | 0.51,3.59 |  | 1.19 | 0.46,3.09 |  |
| **Severe liver disease** | No | 1.36 | 1.03,1.81 | 0.764 | 1.27 | 0.96,1.69 | 0.131 |
|  | Yes | 1.19 | 0.51,2.77 |  | 0.67 | 0.30,1.48 |  |
| **NAFLD** | No | 1.25 | 1.07,1.46 | 0.620 | 1.70 | 1.44,2.01 | 0.318 |
|  | Yes | 1.43 | 0.89,2.30 |  | 1.32 | 0.82,2.13 |  |
| **Gallbladder disease** | No | 1.24 | 1.08,1.41 | 0.262 | 1.19 | 1.04,1.37 | 0.012 |
|  | Yes | 0.92 | 0.56,1.51 |  | 0.63 | 0.40,1.02 |  |
| **Pancreatic disease** | No | 1.31 | 1.06,1.62 | 0.336 | 1.50 | 1.20,1.87 | 0.699 |
|  | Yes | 1.79 | 0.98,3.29 |  | 1.33 | 0.74,2.37 |  |
|  | **Asthma** |  |  |  |  |  |  |
| **GI dysfunction** | No | 1.38 | 1.25,1.53 | 0.821 | 1.21 | 1.09,1.33 | 0.928 |
|  | Yes | 1.34 | 1.09,1.65 |  | 1.19 | 0.97,1.46 |  |
| **PUD** | No | 1.22 | 0.97,1.53 | 0.756 | 0.96 | 0.77,1.20 | 0.965 |
|  | Yes | 1.31 | 0.81,2.14 |  | 0.95 | 0.59,1.52 |  |
| **GERD** | No | 1.40 | 1.28,1.54 | 0.783 | 1.29 | 1.18,1.41 | 0.004 |
|  | Yes | 1.45 | 1.20,1.76 |  | 0.96 | 0.80,1.15 |  |
| **IBD** | No | 1.38 | 0.98,1.94 | 0.753 | 1.24 | 0.88,1.73 | 0.683 |
|  | Yes | 1.21 | 0.58,2.55 |  | 1.05 | 0.51,2.17 |  |
| **Severe liver disease** | No | 1.44 | 1.07,1.93 | 0.281 | 1.29 | 0.96,1.73 | 0.102 |
|  | Yes | 0.96 | 0.50,1.87 |  | 0.72 | 0.38,1.36 |  |
| **NAFLD** | No | 1.19 | 1.01,1.41 | 0.137 | 1.67 | 1.39,2.00 | 0.776 |
|  | Yes | 1.55 | 1.15,2.10 |  | 1.59 | 1.17,2.15 |  |
| **Gallbladder disease** | No | 1.18 | 1.02,1.36 | 0.384 | 1.14 | 0.99,1.31 | 0.698 |
|  | Yes | 1.37 | 1.01,1.86 |  | 1.07 | 0.79,1.44 |  |
| **Pancreatic disease** | No | 1.36 | 1.09,1.70 | 0.964 | 1.55 | 1.23,1.94 | 0.330 |
|  | Yes | 1.35 | 0.83,2.18 |  | 1.19 | 0.74,1.91 |  |
|  | **Dementia** |  |  |  |  |  |  |
| **GI dysfunction** | No | 1.35 | 1.24,1.48 | 0.146 | 1.22 | 1.11,1.33 | 0.026 |
|  | Yes | 2.05 | 1.16,3.60 |  | 0.77 | 0.51,1.17 |  |
| **PUD** | No | 1.25 | 1.02,1.54 | NA | 0.97 | 0.79,1.19 | NA |
|  | Yes | NA | NA |  | NA | NA |  |
| **GERD** | No | 1.41 | 1.3,1.53 | 0.636 | 1.22 | 1.13,1.33 | 0.285 |
|  | Yes | 1.08 | 0.5,2.34 |  | 0.79 | 0.41,1.53 |  |
| **IBD** | No | 1.35 | 0.99,1.85 | NA | 1.23 | 0.90,1.67 | NA |
|  | Yes | NA | NA |  | NA | NA |  |
| **Severe liver disease** | No | 1.33 | 1.01,1.74 | 0.444 | 1.16 | 0.89,1.52 | 0.847 |
|  | Yes | 3.13 | 0.27,36.19 |  | 1.50 | 0.30,7.48 |  |
| **NAFLD** | No | 1.27 | 1.10,1.48 | 0.440 | 1.68 | 1.44,1.97 | 0.072 |
|  | Yes | 0.82 | 0.20,3.43 |  | 0.58 | 0.16,2.08 |  |
| **Gallbladder disease** | No | 1.22 | 1.07,1.38 | 0.303 | 1.14 | 1.00,1.30 | 0.120 |
|  | Yes | 0.61 | 0.22,1.72 |  | 0.48 | 0.19,1.19 |  |
| **Pancreatic disease** | No | 1.37 | 1.12,1.68 | 0.288 | 1.50 | 1.22,1.85 | 0.244 |
|  | Yes | 0.58 | 0.12,2.79 |  | 0.58 | 0.12,2.72 |  |
|  | **Myocardial infarction** |  |  |  |  |  |  |
| **GI dysfunction** | No | 1.36 | 1.24,1.50 | 0.448 | 1.21 | 1.11,1.33 | 0.487 |
|  | Yes | 1.55 | 1.13,2.13 |  | 1.09 | 0.81,1.47 |  |
| **PUD** | No | 1.25 | 1.01,1.55 | 0.692 | 0.99 | 0.80,1.23 | 0.371 |
|  | Yes | 1.08 | 0.54,2.15 |  | 0.72 | 0.37,1.40 |  |
| **GERD** | No | 1.39 | 1.27,1.51 | 0.060 | 1.25 | 1.15,1.36 | 0.072 |
|  | Yes | 1.94 | 1.38,2.72 |  | 0.93 | 0.69,1.27 |  |
| **IBD** | No | 1.20 | 0.86,1.67 | 0.032 | 1.10 | 0.79,1.54 | 0.165 |
|  | Yes | 3.64 | 1.41,9.40 |  | 2.11 | 0.91,4.91 |  |
| **Severe liver disease** | No | 1.18 | 0.88,1.59 | 0.023 | 1.09 | 0.81,1.47 | 0.308 |
|  | Yes | 2.91 | 1.43,5.93 |  | 1.57 | 0.85,2.92 |  |
| **NAFLD** | No | 1.22 | 1.05,1.43 | 0.120 | 1.61 | 1.37,1.90 | 0.436 |
|  | Yes | 1.82 | 1.13,2.93 |  | 1.98 | 1.23,3.19 |  |
| **Gallbladder disease** | No | 1.21 | 1.06,1.38 | 0.975 | 1.16 | 1.01,1.32 | 0.168 |
|  | Yes | 1.21 | 0.74,2.00 |  | 0.82 | 0.51,1.32 |  |
| **Pancreatic disease** | No | 1.43 | 1.16,1.76 | 0.074 | 1.54 | 1.24,1.91 | 0.180 |
|  | Yes | 0.70 | 0.33,1.48 |  | 0.88 | 0.41,1.91 |  |
|  | **Stroke** |  |  |  |  |  |  |
| **GI dysfunction** | No | 1.35 | 1.24,1.48 | 0.152 | 1.21 | 1.11,1.33 | 0.474 |
|  | Yes | 1.79 | 1.22,2.61 |  | 1.05 | 0.74,1.50 |  |
| **PUD** | No | 1.25 | 1.02,1.54 | 0.267 | 1.01 | 0.82,1.24 | 0.040 |
|  | Yes | 0.37 | 0.05,2.88 |  | 0.12 | 0.02,0.86 |  |
| **GERD** | No | 1.42 | 1.31,1.55 | 0.288 | 1.24 | 1.14,1.34 | 0.113 |
|  | Yes | 1.09 | 0.67,1.77 |  | 0.84 | 0.53,1.34 |  |
| **IBD** | No | 1.32 | 0.96,1.81 | 0.564 | 1.21 | 0.88,1.66 | 0.768 |
|  | Yes | 1.99 | 0.49,8.14 |  | 0.99 | 0.27,3.57 |  |
| **Severe liver disease** | No | 1.32 | 1.00,1.74 | 0.612 | 1.15 | 0.87,1.51 | 0.661 |
|  | Yes | 1.77 | 0.61,5.16 |  | 1.47 | 0.55,3.90 |  |
| **NAFLD** | No | 1.26 | 1.08,1.46 | 0.707 | 1.63 | 1.39,1.92 | 0.678 |
|  | Yes | 1.47 | 0.75,2.89 |  | 1.96 | 0.96,3.98 |  |
| **Gallbladder disease** | No | 1.20 | 1.05,1.37 | 0.607 | 1.13 | 0.99,1.29 | 0.851 |
|  | Yes | 1.47 | 0.77,2.80 |  | 1.08 | 0.58,2.01 |  |
| **Pancreatic disease** | No | 1.34 | 1.09,1.65 | 0.522 | 1.53 | 1.23,1.89 | 0.195 |
|  | Yes | 1.84 | 0.73,4.65 |  | 0.87 | 0.38,1.96 |  |
|  | **COPD** |  |  |  |  |  |  |
| **GI dysfunction** | No | 1.36 | 1.24,1.49 | 0.515 | 1.24 | 1.13,1.36 | 0.125 |
|  | Yes | 1.51 | 1.12,2.03 |  | 0.99 | 0.75,1.30 |  |
| **PUD** | No | 1.23 | 1.00,1.53 | 0.883 | 0.99 | 0.80,1.23 | 0.359 |
|  | Yes | 1.16 | 0.56,2.41 |  | 0.71 | 0.35,1.41 |  |
| **GERD** | No | 1.40 | 1.29,1.52 | 0.513 | 1.27 | 1.17,1.39 | 0.003 |
|  | Yes | 1.57 | 1.12,2.21 |  | 0.79 | 0.58,1.08 |  |
| **IBD** | No | 1.25 | 0.90,1.73 | 0.097 | 1.17 | 0.84,1.63 | 0.751 |
|  | Yes | 2.95 | 1.09,7.99 |  | 1.34 | 0.58,3.09 |  |
| **Severe liver disease** | No | 1.31 | 0.98,1.75 | 0.747 | 1.18 | 0.88,1.58 | 0.821 |
|  | Yes | 1.48 | 0.75,2.92 |  | 1.08 | 0.57,2.06 |  |
| **NAFLD** | No | 1.21 | 1.03,1.42 | 0.177 | 1.59 | 1.35,1.88 | 0.423 |
|  | Yes | 1.65 | 1.09,2.50 |  | 1.91 | 1.26,2.90 |  |
| **Gallbladder disease** | No | 1.21 | 1.06,1.38 | 0.891 | 1.15 | 1.01,1.32 | 0.225 |
|  | Yes | 1.16 | 0.74,1.83 |  | 0.86 | 0.56,1.33 |  |
| **Pancreatic disease** | No | 1.42 | 1.15,1.75 | 0.116 | 1.56 | 1.26,1.94 | 0.097 |
|  | Yes | 0.77 | 0.37,1.58 |  | 0.82 | 0.40,1.69 |  |
|  | **Diabetes** |  |  |  |  |  |  |
| **GI dysfunction** | No | 1.34 | 1.21,1.47 | 0.221 | 1.18 | 1.07,1.30 | 0.448 |
|  | Yes | 1.56 | 1.25,1.95 |  | 1.30 | 1.05,1.60 |  |
| **PUD** | No | 1.34 | 1.07,1.66 | 0.048 | 1.10 | 0.89,1.36 | 0.004 |
|  | Yes | 0.69 | 0.36,1.30 |  | 0.41 | 0.22,0.77 |  |
| **GERD** | No | 1.42 | 1.30,1.54 | 0.767 | 1.30 | 1.19,1.41 | <0.001 |
|  | Yes | 1.38 | 1.07,1.78 |  | 0.80 | 0.63,1.01 |  |
| **IBD** | No | 1.36 | 0.97,1.90 | 0.910 | 1.18 | 0.84,1.65 | 0.789 |
|  | Yes | 1.32 | 0.61,2.85 |  | 1.35 | 0.63,2.90 |  |
| **Severe liver disease** | No | 1.06 | 0.75,1.50 | 0.031 | 1.03 | 0.72,1.47 | 0.321 |
|  | Yes | 2.00 | 1.29,3.11 |  | 1.37 | 0.91,2.06 |  |
| **NAFLD** | No | 1.20 | 1.01,1.43 | 0.365 | 1.79 | 1.48,2.15 | 0.096 |
|  | Yes | 1.41 | 1.06,1.88 |  | 1.34 | 1.01,1.79 |  |
| **Gallbladder disease** | No | 1.15 | 1.00,1.32 | 0.091 | 1.11 | 0.96,1.28 | 0.835 |
|  | Yes | 1.53 | 1.12,2.10 |  | 1.14 | 0.84,1.54 |  |
| **Pancreatic disease** | No | 1.32 | 1.06,1.65 | 0.672 | 1.62 | 1.29,2.04 | 0.058 |
|  | Yes | 1.47 | 0.92,2.34 |  | 0.99 | 0.64,1.55 |  |
|  | **BMI** |  |  |  |  |  |  |
| **GI dysfunction** | <30 | 1.36 | 1.22,1.52 | 0.849 | 1.16 | 1.04,1.29 | 0.208 |
|  | >=30 | 1.39 | 1.19,1.62 |  | 1.30 | 1.12,1.52 |  |
| **PUD** | <30 | 1.44 | 1.12,1.85 | 0.032 | 1.04 | 0.81,1.32 | 0.221 |
|  | >=30 | 0.88 | 0.61,1.28 |  | 0.79 | 0.54,1.14 |  |
| **GERD** | <30 | 1.41 | 1.28,1.56 | 0.844 | 1.23 | 1.12,1.36 | 0.608 |
|  | >=30 | 1.39 | 1.20,1.61 |  | 1.18 | 1.02,1.36 |  |
| **IBD** | <30 | 1.34 | 0.94,1.92 | 0.891 | 1.28 | 0.89,1.83 | 0.554 |
|  | >=30 | 1.42 | 0.76,2.64 |  | 1.04 | 0.57,1.89 |  |
| **Severe liver disease** | <30 | 1.31 | 0.92,1.86 | 0.859 | 1.26 | 0.88,1.80 | 0.468 |
|  | >=30 | 1.37 | 0.91,2.08 |  | 1.03 | 0.69,1.55 |  |
| **NAFLD** | <30 | 1.21 | 0.98,1.50 | 0.721 | 1.63 | 1.30,2.05 | 0.936 |
|  | >=30 | 1.28 | 1.04,1.56 |  | 1.61 | 1.30,1.99 |  |
| **Gallbladder disease** | <30 | 1.14 | 0.96,1.35 | 0.437 | 1.02 | 0.86,1.21 | 0.119 |
|  | >=30 | 1.27 | 1.04,1.55 |  | 1.26 | 1.03,1.54 |  |
| **Pancreatic disease** | <30 | 1.16 | 0.89,1.50 | 0.045 | 1.35 | 1.03,1.76 | 0.301 |
|  | >=30 | 1.77 | 1.28,2.45 |  | 1.68 | 1.22,2.32 |  |
|  | **Deprivation Index** |  |  |  |  |  |  |
| **GI dysfunction** | <mean | 1.36 | 1.20,1.53 | 0.732 | 1.22 | 1.08,1.37 | 0.739 |
|  | >=mean | 1.40 | 1.22,1.60 |  | 1.18 | 1.03,1.34 |  |
| **PUD** | <mean | 1.08 | 0.81,1.45 | 0.236 | 0.89 | 0.66,1.19 | 0.496 |
|  | >=mean | 1.39 | 1.04,1.86 |  | 1.02 | 0.77,1.35 |  |
| **GERD** | <mean | 1.37 | 1.23,1.53 | 0.558 | 1.16 | 1.04,1.29 | 0.182 |
|  | >=mean | 1.44 | 1.28,1.63 |  | 1.29 | 1.15,1.46 |  |
| **IBD** | <mean | 1.50 | 0.99,2.28 | 0.462 | 1.22 | 0.81,1.83 | 0.911 |
|  | >=mean | 1.19 | 0.75,1.88 |  | 1.17 | 0.74,1.86 |  |
| **Severe liver disease** | <mean | 1.24 | 0.83,1.84 | 0.622 | 1.03 | 0.70,1.51 | 0.410 |
|  | >=mean | 1.41 | 0.98,2.04 |  | 1.28 | 0.89,1.85 |  |
| **NAFLD** | <mean | 1.14 | 0.92,1.41 | 0.246 | 1.44 | 1.15,1.81 | 0.151 |
|  | >=mean | 1.36 | 1.11,1.67 |  | 1.82 | 1.47,2.25 |  |
| **Gallbladder disease** | <mean | 1.17 | 0.98,1.39 | 0.661 | 1.01 | 0.84,1.20 | 0.081 |
|  | >=mean | 1.24 | 1.03,1.49 |  | 1.27 | 1.05,1.54 |  |
| **Pancreatic disease** | <mean | 1.10 | 0.82,1.47 | 0.056 | 1.29 | 0.95,1.75 | 0.273 |
|  | >=mean | 1.63 | 1.24,2.15 |  | 1.63 | 1.23,2.16 |  |

GI, gastrointestinal; GERD, gastroesophageal reflux disease; PUD, peptic ulcer disease; IBD, inflammatory bowel disease; NAFLD, non-alcohol fatty liver disease;

BMI: body mass index; COPD: chronic obstructive pulmonary disease; HR: hazard ratio; CI: confidence interval; Pi: P value for interaction.

Weighted HRs after IPTW and 95% CIs are presented.

Table S17. Hazard ratio of digestive outcomes in COVID-19 group, the contemporary and historical comparison by sex

| **Outcome** | **Subgroup** | **COVID-19 vs Contemporary comparison** | | | **COVID-19 vs Historical comparison** | | |
| --- | --- | --- | --- | --- | --- | --- | --- |
|  |  | **HR** | **95% CI** | **Pi** | **HR** | **95% CI** | **Pi** |
| **GI dysfunction** | Male | 1.48 | 1.30,1.69 | 0.142 | 1.29 | 1.13,1.46 | 0.160 |
|  | Female | 1.29 | 1.15,1.46 |  | 1.13 | 1.00,1.28 |  |
| **PUD** | Male | 1.28 | 0.97,1.69 | 0.700 | 0.92 | 0.70,1.21 | 0.679 |
|  | Female | 1.17 | 0.86,1.59 |  | 1.00 | 0.74,1.36 |  |
| **GERD** | Male | 1.41 | 1.25,1.59 | 0.963 | 1.22 | 1.08,1.38 | 0.987 |
|  | Female | 1.42 | 1.27,1.58 |  | 1.22 | 1.10,1.36 |  |
| **IBD** | Male | 1.56 | 1.02,2.38 | 0.371 | 1.24 | 0.82,1.88 | 0.795 |
|  | Female | 1.17 | 0.74,1.84 |  | 1.15 | 0.73,1.81 |  |
| **Severe liver disease** | Male | 1.50 | 1.08,2.08 | 0.270 | 1.28 | 0.93,1.77 | 0.350 |
|  | Female | 1.07 | 0.67,1.73 |  | 0.96 | 0.60,1.55 |  |
| **NAFLD** | Male | 1.16 | 0.94,1.43 | 0.275 | 1.65 | 1.32,2.06 | 0.967 |
|  | Female | 1.38 | 1.12,1.70 |  | 1.65 | 1.33,2.05 |  |
| **Gallbladder disease** | Male | 1.24 | 1.02,1.51 | 0.793 | 1.13 | 0.93,1.38 | 0.926 |
|  | Female | 1.19 | 1.01,1.42 |  | 1.12 | 0.95,1.33 |  |
| **Pancreatic disease** | Male | 1.11 | 0.82,1.48 | 0.057 | 1.25 | 0.93,1.70 | 0.150 |
|  | Female | 1.63 | 1.24,2.14 |  | 1.69 | 1.28,2.24 |  |

GI, gastrointestinal;GERD, gastroesophageal reflux disease; PUD, peptic ulcer disease; IBD, inflammatory bowel disease; NAFLD, non-alcohol fatty liver disease;

HR: hazard ratio; CI: confidence interval; Pi: P value for interaction.

Weighted HRs after IPTW and 95% CIs are presented.

Table S18. Baseline characteristics of COVID-19 group and historical comparisons before weighting

| Characteristics | COVID-19 group  (n = 112 311） | Historical comparisons  (n = 370 979) | SMD |
| --- | --- | --- | --- |
| Age, mean(SD), years | 54.43 (8.19) | 56.94 (7.97) | 0.312 |
| Sex, female, n(%) | 50 816 (45.2) | 166 904 (45.0) | 0.005 |
| Ethnicity, White, n(%) | 106 315 (94.7) | 350 562 (94.5) | 0.007 |
| Household income |  |  | 0.201 |
| <18 000, n(%) | 19 465 (17.3) | 87 290 (23.5) |  |
| 18 000-30 999, n(%) | 26 102 (23.2) | 97 269 (26.2) |  |
| 31 000-51 999, n(%) | 31 853 (28.4) | 94 903 (25.6) |  |
| 52 000-100 000, n(%) | 27 297 (24.3) | 71 710 (19.3) |  |
| >100 000, n(%) | 7 594 (6.8) | 19 807 (5.3) |  |
| Deprivation index, mean(SD) | -1.38 (3.00) | -1.30 (3.10) | 0.028 |
| BMI, mean(SD), kg/m^2^ | 27.47 (4.82) | 27.39 (4.76) | 0.018 |
| Alcohol consumption |  |  | 0.104 |
| Daily or almost daily, n(%) | 21 925 (19.5) | 75 808 (20.4) |  |
| Three or four times a week, n(%) | 28 027 (25.0) | 84 054 (22.7) |  |
| Once or twice a week, n(%) | 30 734 (27.4) | 94 503 (25.5) |  |
| One to three times a month, n(%) | 12 790 (11.4) | 41 435 (11.2) |  |
| Special occasions only or never, n(%) | 11 298 (10.1) | 44 263 (11.9) |  |
| Never, n(%) | 7 537 (6.7) | 30 916 (8.3) |  |
| Smoking status |  |  | 0.062 |
| Never smoker, n(%) | 63 505 (56.5) | 204 121 (55.0) |  |
| Previous smoker, n(%) | 38 875 (34.6) | 127 264 (34.3) |  |
| Current smoker, n(%) | 9 931 (8.8) | 39 594 (10.7) |  |
| Physical activity, mean(SD), MET minutes/week | 2536.46 (2603.69) | 2683.22 (2740.86) | 0.055 |
| Comorbidities |  |  |  |
| Hypertension, n(%) | 38 023 (33.9) | 139 121 (37.5) | 0.076 |
| Diabetes, n(%) | 8 384 (7.5) | 30 043 (8.1) | 0.024 |
| Renal failure, n(%) | 4 646 (4.1) | 17 084 (4.6) | 0.023 |
| Myocardial infarction, n(%) | 4 557 (4.1) | 17 795 (4.8) | 0.036 |
| Stroke, n(%) | 2 706 (2.4) | 11 228 (3.0) | 0.038 |
| COPD, n(%) | 4 377 (3.9) | 17 197 (4.6) | 0.037 |
| Asthma, n(%) | 17 231 (15.3) | 50 073 (13.5) | 0.053 |
| Heart failure, n(%) | 2 356 (2.1) | 10 127 (2.7) | 0.041 |
| Dementia, n(%) | 1 060 (0.9) | 3 788 (1.0) | 0.008 |
| Recent hospital admissions, mean(SD) | 1.0(1.9) | 0.9(1.7) | 0.071 |
| History of previous digestive diseases, n(%) | 37 375 (33.3) | 109 941 (29.6) | 0.079 |

SMD: standard mean difference; BMI: body mass index; MET: metabolic equivalent of task; COPD: chronic obstructive pulmonary disease; SD: standard deviation

Table S19. Baseline characteristics of COVID-19 group and historical comparisons after weighting

| Characteristics | COVID-19 group  (n = 112 311) | Historical comparisons  (n = 370 979) | SMD |
| --- | --- | --- | --- |
| Age, mean(SD), years | 56.3(8.1) | 56.4(8.1) | 0.003 |
| Sex, female, n(%) | 61 322(54.6) | 203 667(54.9) | 0.007 |
| Ethnicity, White, n(%) | 106 246(94.6) | 350 575(94.5) | 0.001 |
| Household income |  |  | 0.005 |
| <18 000, n(%) | 24 596(21.9) | 81 986(22.1) |  |
| 18 000-30 999, n(%) | 28 639(25.5) | 94 600(25.5) |  |
| 31 000-51 999, n(%) | 29 538(26.3) | 97 196(26.2) |  |
| 52 000-100 000, n(%) | 23 248(20.7) | 76 051(20.5) |  |
| >100 000, n(%) | 6 402(5.7) | 21 146(5.7) |  |
| Deprivation index, mean(SD) | -1.3(3.0) | -1.3(3.1) | 0.003 |
| BMI, mean(SD), kg/m^2^ | 27.4(4.7) | 27.4(4.8) | 0.002 |
| Alcohol consumption |  |  | 0.005 |
| Daily or almost daily, n(%) | 22 911(20.4) | 74 938(20.2) |  |
| Three or four times a week, n(%) | 26 168(23.3) | 86 067(23.2) |  |
| Once or twice a week, n(%) | 29 089(25.9) | 96 084(25.9) |  |
| One to three times a month, n(%) | 12 467(11.1) | 41 550(11.2) |  |
| Special occasions only or never, n(%) | 12 803(11.4) | 42 663(11.5) |  |
| Never, n(%) | 8 985(8) | 29 678(8) |  |
| Smoking status |  |  | 0.002 |
| Never smoker, n(%) | 62 220(55.4) | 205 522(55.4) |  |
| Previous smoker, n(%) | 38 635(34.4) | 127 617(34.4) |  |
| Current smoker, n(%) | 11 456(10.2) | 37 840(10.2) |  |
| Physical activity, mean(SD), MET minutes/week | 2645.2(2702.9) | 2648.5(2711.0) | 0.002 |
| Comorbidities |  |  |  |
| Hypertension, n(%) | 41 106(36.6) | 136 149(36.7) | <0.001 |
| Diabetes, n(%) | 8 873(7.9) | 29 678(8) | <0.001 |
| Renal failure, n(%) | 5 054(4.5) | 16 694(4.5) | 0.001 |
| Myocardial infarction, n(%) | 5 166(4.6) | 17 065(4.6) | <0.001 |
| Stroke, n(%) | 3 032(2.7) | 10 758(2.9) | <0.001 |
| COPD, n(%) | 5 054(4.5) | 16 694(4.5) | <0.001 |
| Asthma, n(%) | 15 724(14) | 51 566(13.9) | 0.001 |
| Heart failure, n(%) | 2 920(2.6) | 9 645(2.6) | 0.011 |
| Dementia, n(%) | 1 123(1) | 3 710(1) | 0.003 |
| Recent hospital admissions, mean(SD) | 0.9(1.7) | 0.9(1.8) | 0.007 |
| History of previous digestive diseases, n(%) | 34 030(30.3) | 113 149(30.5) | 0.003 |

SMD: standard mean difference; BMI: body mass index; MET: metabolic equivalent of task; COPD: chronic obstructive pulmonary disease; SD: standard deviation

Table S20. Baseline characteristics of COVID-19 group and historical comparisons by severity of COVID-19 before weighting

| Characteristics | Non-hospitalized COVID  (n= 104 201) | Hospitalized COVID  (n= 7 523) | Severe COVID  (n= 588) | Historical comparisons  (n = 370 979) | SMD | | |
| --- | --- | --- | --- | --- | --- | --- | --- |
|  |  |  |  |  | Non-hospitalized COVID and historical comparisons | Hospitalized COVID and historical comparisons | Severe COVID and historical comparisons |
| Age, mean(SD), years | 54.02(8.09) | 59.84(7.55) | 57.78(7.99) | 56.94(7.97) | 0.365 | 0.374 | 0.105 |
| Sex, female, n(%) | 57 959(55.6) | 3 324(44.2) | 212(36.1) | 166 904 (45.0) | 0.012 | 0.218 | 0.388 |
| Ethnicity, White, n(%) | 98 873(94.9) | 6 951(92.4) | 492(83.7) | 350 562 (94.5) |  |  |  |
| Household income |  |  |  |  | 0.244 | 0.322 | 0.378 |
| <18 000, n(%) | 16 497(15.8) | 2 741(36.4) | 227(38.6) | 87 290 (23.5) |  |  |  |
| 18 000-30 999, n(%) | 23 945(23.0) | 2 003(26.6) | 154(26.2) | 97 269 (26.2) |  |  |  |
| 31 000-51 999, n(%) | 30 165(28.9) | 1 567(20.8) | 122(20.7) | 94 903 (25.6) |  |  |  |
| 52 000-100 000, n(%) | 26 266(25.2) | 961(12.8) | 70(11.9) | 71 710 (19.3) |  |  |  |
| >100 000, n(%) | 7 328(7.0) | 251(3.3) | 15(2.6) | 19 807 ( 5.3) |  |  |  |
| Deprivation index, mean(SD) | -1.46(2.95) | -0.43(3.40) | 0.16(3.54) | -1.30 (3.10) | 0.054 | 0.268 | 0.437 |
| BMI, mean(SD), kg/m^2^ | 27.33(4.72) | 29.23(5.63) | 30.61(5.78) | 27.39 (4.76) | 0.012 | 0.353 | 0.608 |
| Alcohol consumption |  |  |  |  | 0.129 | 0.204 | 0.312 |
| Daily or almost daily, n(%) | 20 416(19.6) | 1 414(18.8) | 96(16.3) | 75 808 (20.4) |  |  |  |
| Three or four times a week, n(%) | 26 582(25.5) | 1353(18.0) | 92(15.6) | 84 054 (22.7) |  |  |  |
| Once or twice a week, n(%) | 28 762(27.6) | 1 825(24.3) | 147(25.0) | 94 503 (25.5) |  |  |  |
| One to three times a month, n(%) | 11 927(11.4) | 799(10.6) | 64(10.9) | 41 435 (11.2) |  |  |  |
| Special occasions only or never, n(%) | 10 034(9.6) | 1 168(15.5) | 96(16.3) | 44 263 (11.9) |  |  |  |
| Never, n(%) | 6 480(6.2) | 964(12.8) | 93(15.8) | 30 916 ( 8.3) |  |  |  |
| Smoking status |  |  |  |  | 0.080 | 0.217 | 0.252 |
| Never smoker, n(%) | 59 921(57.5) | 3 335(44.3) | 250(42.5) | 204 121 (55.0) |  |  |  |
| Previous smoker, n(%) | 35 500(34.1) | 3 115(41.4) | 260(44.2) | 127 264 (34.3) |  |  |  |
| Current smoker, n(%) | 8 780(8.4) | 1 073(14.3) | 78(13.3) | 39 594 (10.7) |  |  |  |
| Physical activity, mean(SD), MET minutes/week | 2529.22  (2585.74) | 2623.26  (2813.78) | 2707.49  (2935.05) | 2683.22 (2740.86) | 0.058 | 0.022 | 0.009 |
| Comorbidities |  |  |  |  |  |  |  |
| Hypertension, n(%) | 33 161(31.8) | 4 523(60.1) | 339(57.7) | 139 121 (37.5) | 0.12 | 0.465 | 0.412 |
| Diabetes, n(%) | 6 579(6.3) | 1 678(22.3) | 127(21.6) | 30 043 ( 8.1) | 0.069 | 0.404 | 0.387 |
| Renal failure, n(%) | 3 515(3.4) | 1 050(14.0) | 81(13.8) | 17 084 ( 4.6) | 0.063 | 0.327 | 0.322 |
| Myocardial infarction, n(%) | 3 599(3.5) | 890(11.8) | 68(11.6) | 17 795 ( 4.8) | 0.068 | 0.257 | 0.249 |
| Stroke, n(%) | 2 056(2.0) | 623(8.3) | 27(4.6) | 11 228 ( 3.0) | 0.068 | 0.229 | 0.082 |
| COPD, n(%) | 3 174(3.0) | 1 123(14.9) | 80(13.6) | 17 197 ( 4.6) | 0.083 | 0.352 | 0.315 |
| Asthma, n(%) | 15 561(14.9) | 1 534(20.4) | 136(23.1) | 50 073 (13.5) | 0.041 | 0.185 | 0.251 |
| Heart failure, n(%) | 1 607(1.5) | 714(9.5) | 35(6.0) | 10 127 ( 2.7) | 0.082 | 0.285 | 0.159 |
| Dementia, n(%) | 732(0.7) | 321(4.3) | 7(1.2) | 3 788 ( 1.0) | 0.034 | 0.203 | 0.016 |
| Recent hospital admissions, mean(SD) | 0.8(1.5) | 3.5(4.0) | 2.1(2.8) | 0.9(1.7) | 0.036 | 0.854 | 0.543 |
| History of previous digestive diseases, n(%) | 33 024(31.7) | 4 085(54.3) | 266(45.2) | 109 941 (29.6) | 0.045 | 0.516 | 0.327 |

SMD: standard mean difference; BMI: body mass index; MET: metabolic equivalent of task; COPD: chronic obstructive pulmonary disease; SD: standard deviation

Table S21. Baseline characteristics of COVID-19 group and historical comparisons by severity of COVID-19 after weighting

| Characteristics | Non-hospitalized COVID  (n= 104 201) | Hospitalized COVID  (n= 7 523) | Severe COVID  (n= 588) | Historical comparisons  (n = 370 979) | SMD | | |
| --- | --- | --- | --- | --- | --- | --- | --- |
|  |  |  |  |  | Non-hospitalized COVID and historical comparisons | Hospitalized COVID and historical comparisons | Severe COVID and historical comparisons |
| Age, mean(SD), years | 56.3(8.0) | 57.0(8.3) | 57.6(7.6) | 56.4(8.1) | 0.004 | 0.005 | 0.082 |
| Sex, female, n(%) | 57 102(54.8) | 3 972(52.8) | 288(49) | 203 667(54.9) | 0.006 | 0.039 | 0.119 |
| Ethnicity, White, n(%) | 98 574(94.6) | 7 072(94) | 550(93.5) | 350 575(94.5) | 0.001 | 0.019 | 0.041 |
| Household income |  |  |  |  | 0.007 | 0.029 | 0.087 |
| <18 000, n(%) | 22 507(21.6) | 1 873(24.9) | 151(25.6) | 81 986(22.1) |  |  |  |
| 18 000-30 999, n(%) | 26 467(25.4) | 1 948(25.9) | 167(28.4) | 94 600(25.5) |  |  |  |
| 31 000-51 999, n(%) | 27 509(26.4) | 1 933(25.7) | 142(24.1) | 97 196(26.2) |  |  |  |
| 52 000-100 000, n(%) | 21 674(20.8) | 1 384(18.4) | 100(17) | 76 051(20.5) |  |  |  |
| >100 000, n(%) | 6 044(5.8) | 391(5.2) | 29(4.9) | 21 146(5.7) |  |  |  |
| Deprivation index, mean(SD) | -1.46(2.95) | -0.43(3.40) | 0.16(3.54) | -1.3(3.1) | 0.054 | 0.268 | 0.437 |
| BMI, mean(SD), kg/m^2^ | 27.33(4.72) | 29.23(5.63) | 30.61(5.78) | 27.4(4.8) | 0.012 | 0.353 | 0.608 |
| Alcohol consumption |  |  |  |  | 0.007 | 0.047 | 0.163 |
| Daily or almost daily, n(%) | 21 257(20.4) | 1 550(20.6) | 105(17.9) | 74 938(20.2) |  |  |  |
| Three or four times a week, n(%) | 24 383(23.4) | 1 610(21.4) | 116(19.8) | 86 067(23.2) |  |  |  |
| Once or twice a week, n(%) | 26 988(25.9) | 1 836(24.4) | 146(24.9) | 96 084(25.9) |  |  |  |
| One to three times a month, n(%) | 11 566(11.1) | 895(11.9) | 82(13.9) | 41 550(11.2) |  |  |  |
| Special occasions only or never, n(%) | 11 775(11.3) | 955(12.7) | 67(11.4) | 42 663(11.5) |  |  |  |
| Never, n(%) | 8 232(7.9) | 677(9) | 71(12) | 29 678(8) |  |  |  |
| Smoking status |  |  |  |  | 0.006 | 0.046 | 0.065 |
| Never smoker, n(%) | 58 040(55.7) | 3 950(52.5) | 320(54.5) | 205 522(55.4) |  |  |  |
| Previous smoker, n(%) | 35 741(34.3) | 2 723(36.2) | 193(32.8) | 127 617(34.4) |  |  |  |
| Current smoker, n(%) | 10 420(10.0) | 850(11.3) | 75(12.7) | 37 840(10.2) |  |  |  |
| Physical activity, mean(SD), MET minutes/week | 2643.0(2695.1) | 2637.9(2707.0) | 2639.3(2714.8) | 2648.5(2711.0) | 0.002 | 0.016 | 0.016 |
| Comorbidities |  |  |  |  |  |  |  |
| Hypertension, n(%) | 37 721(36.2) | 3 024(40.2) | 259(44.1) | 136 149(36.7) | 0.002 | 0.044 | 0.134 |
| Diabetes, n(%) | 8 023(7.7) | 722(9.6) | 68(11.6) | 29 678(8) | 0.002 | 0.040 | 0.117 |
| Renal failure, n(%) | 4 481(4.3) | 451(6) | 36(6.1) | 16 694(4.5) | 0.001 | 0.048 | 0.066 |
| Myocardial infarction, n(%) | 4 689(4.5) | 436(5.8) | 39(6.7) | 17 065(4.6) | 0.001 | 0.038 | 0.083 |
| Stroke, n(%) | 2 918(2.8) | 278(3.7) | 23(3.9) | 10 758(2.9) | 0.002 | 0.032 | 0.049 |
| COPD, n(%) | 4 481(4.3) | 474(6.3) | 40(6.8) | 16 694(4.5) | 0.001 | 0.059 | 0.092 |
| Asthma, n(%) | 14 380(13.8) | 1 106(14.7) | 96(16.4) | 51 566(13.9) | <0.001 | 0.030 | 0.081 |
| Heart failure, n(%) | 2 605(2.5) | 308(4.1) | 31(5.2) | 9 645(2.6) | 0.001 | 0.063 | 0.128 |
| Dementia, n(%) | 1 042(1) | 120(1.6) | 3(0.5) | 3 710(1) | 0.003 | 0.039 | 0.060 |
| Recent hospital admissions, mean(SD) | 0.9(1.6) | 1.4(1.9) | 1.3(1.8) | 0.9(1.8) | 0.001 | 0.188 | 0.232 |
| History of previous digestive diseases, n(%) | 31 365(30.1) | 2 197(29.2) | 199(33.8) | 113 149(30.5) | <0.001 | 0.023 | 0.090 |

SMD: standard mean difference; BMI: body mass index; MET: metabolic equivalent of task; COPD: chronic obstructive pulmonary disease; SD: standard deviation

Table S22. Baseline characteristics of COVID-19 group and historical comparisons in the sensitive analysis restricting to the period before vaccination was available before weighting.

| Characteristics | COVID-19 group  (n = 8 431) | Historical comparisons  (n = 370 979) | SMD |
| --- | --- | --- | --- |
| Age, mean(SD), years | 54.39(8.82) | 56.94(7.97) | 0.304 |
| Sex, female, n(%) | 4 280(50.8) | 204 075(55.0) | 0.085 |
| Ethnicity, White, n(%) | 7 771(92.2) | 350 562(94.5) | 0.093 |
| Household income |  |  | 0.064 |
| <18 000, n(%) | 2 147(25.5) | 87 290(23.5) |  |
| 18 000-30 999, n(%) | 2 119(25.1) | 97 269(26.2) |  |
| 31 000-51 999, n(%) | 2 236(26.5) | 94 903(25.6) |  |
| 52 000-100 000, n(%) | 1 546(18.3) | 71 710(19.3) |  |
| >100 000, n(%) | 383(4.5) | 19 807(5.3) |  |
| Deprivation index, mean(SD) | -0.73(3.27) | -1.30(3.10) | 0.178 |
| BMI, mean(SD), kg/m^2^ | 28.43(5.07) | 27.39(4.76) | 0.211 |
| Alcohol consumption |  |  | 0.115 |
| Daily or almost daily, n(%) | 1 408(16.7) | 75 808(20.4) |  |
| Three or four times a week, n(%) | 1 843(21.9) | 84 054(22.7) |  |
| Once or twice a week, n(%) | 2 394(28.4) | 94 503(25.5) |  |
| One to three times a month, n(%) | 965(11.4) | 41 435(11.2) |  |
| Special occasions only or never, n(%) | 1 004(11.9) | 44 263(11.9) |  |
| Never, n(%) | 817(9.7) | 30 916(8.3) |  |
| Smoking status |  |  | 0.076 |
| Never smoker, n(%) | 4 322(51.3) | 204 121(55.0) |  |
| Previous smoker, n(%) | 3 115(36.9) | 127 264(34.3) |  |
| Current smoker, n(%) | 994(11.8) | 39 594(10.7) |  |
| Physical activity, mean(SD), MET minutes/week | 2734.76(2828.44) | 2683.22(2740.86) | 0.019 |
| Comorbidities |  |  |  |
| Hypertension, n(%) | 3 411(40.5) | 139 121(37.5) | 0.061 |
| Diabetes, n(%) | 1 005(11.9) | 30 043(8.1) | 0.128 |
| Renal failure, n(%) | 573(6.8) | 17 084(4.6) | 0.095 |
| Myocardial infarction, n(%) | 547(6.5) | 17 795(4.8) | 0.073 |
| Stroke, n(%) | 398(4.7) | 11 228(3.0) | 0.088 |
| COPD, n(%) | 616(7.3) | 17 197(4.6) | 0.113 |
| Asthma, n(%) | 1 382(16.4) | 50 073(13.5) | 0.081 |
| Heart failure, n(%) | 385(4.6) | 10 127(2.7) | 0.098 |
| Dementia, n(%) | 345(4.1) | 3 788(1.0) | 0.195 |
| Recent hospital admissions, mean(SD) | 1.6(2.9) | 0.9(1.7) | 0.310 |
| History of previous digestive diseases, n(%) | 3 286(39.0) | 109 941(29.6) | 0.198 |

SMD: standard mean difference; BMI: body mass index; MET: metabolic equivalent of task; COPD: chronic obstructive pulmonary disease; SD: standard deviation

Table S23. Baseline characteristics of COVID-19 group and historical comparisons in the sensitive analysis restricting to the period before vaccination was available after weighting.

| Characteristics | COVID-19 group  (n = 8 431) | Historical comparisons  (n = 370 979) | SMD |
| --- | --- | --- | --- |
| Age, mean(SD), years | 56.1(8.6) | 56.4(8.1) | 0.028 |
| Sex, female, n(%) | 4 527(53.7) | 204038(55) | 0.025 |
| Ethnicity, White, n(%) | 7 950(94.3) | 350575(94.5) | 0.012 |
| Household income |  |  | 0.025 |
| <18 000, n(%) | 1 939(23) | 81986(22.1) |  |
| 18 000-30 999, n(%) | 2 099(24.9) | 94600(25.5) |  |
| 31 000-51 999, n(%) | 2 192(26) | 97196(26.2) |  |
| 52 000-100 000, n(%) | 1 720(20.4) | 76051(20.5) |  |
| >100 000, n(%) | 481(5.7) | 21146(5.7) |  |
| Deprivation index, mean(SD) | -1.2(3.1) | -1.3(3.1) | 0.029 |
| BMI, mean(SD), kg/m^2^ | 27.6(4.6) | 27.4(4.8) | 0.048 |
| Alcohol consumption |  |  | 0.014 |
| Daily or almost daily, n(%) | 1 728(20.5) | 74938(20.2) |  |
| Three or four times a week, n(%) | 1 931(22.9) | 86067(23.2) |  |
| Once or twice a week, n(%) | 2 158(25.6) | 96084(25.9) |  |
| One to three times a month, n(%) | 944(11.2) | 41550(11.2) |  |
| Special occasions only or never, n(%) | 986(11.7) | 42663(11.5) |  |
| Never, n(%) | 683(8.1) | 29678(8) |  |
| Smoking status |  |  | 0.029 |
| Never smoker, n(%) | 4 586(54.4) | 205522(55.4) |  |
| Previous smoker, n(%) | 2 917(34.6) | 127617(34.4) |  |
| Current smoker, n(%) | 936(11.1) | 37840(10.2) |  |
| Physical activity, mean(SD), MET minutes/week | 2665.8(2691.2) | 2648.3(2710.2) | 0.006 |
| Comorbidities |  |  |  |
| Hypertension, n(%) | 3 178(37.7) | 136149(36.7) | 0.023 |
| Diabetes, n(%) | 725(8.6) | 29307(7.9) | 0.022 |
| Renal failure, n(%) | 379(4.5) | 16694(4.5) | 0.001 |
| Myocardial infarction, n(%) | 405(4.8) | 17065(4.6) | 0.010 |
| Stroke, n(%) | 278(3.3) | 10758(2.9) | 0.024 |
| COPD, n(%) | 413(4.9) | 16694(4.5) | 0.022 |
| Asthma, n(%) | 1 214(14.4) | 51566(13.9) | 0.013 |
| Heart failure, n(%) | 236(2.8) | 9645(2.6) | 0.013 |
| Dementia, n(%) | 101(1.2) | 3710(1.0) | 0.017 |
| Recent hospital admissions, mean(SD) | 0.9 (1.8) | 1.0 (1.8) | 0.059 |
| History of previous digestive diseases, n(%) | 3 263(38.7) | 110 552(29.8) | 0.023 |

SMD: standard mean difference; BMI: body mass index; MET: metabolic equivalent of task; COPD: chronic obstructive pulmonary disease; SD: standard deviation

Table S24. Hazard ratio of digestive outcomes in COVID-19 group and the historical comparison by severity of COVID-19

| Outcome | HR (95% CI) | P value |
| --- | --- | --- |
| GI dysfunction |  |  |
| Non-hospitalized COVID | 1.03(0.93,1.14) | 0.549 |
| Hospitalized COVID | 3.33(2.68,4.13) | **<0.001** |
| Severe COVID | 2.74(1.42,5.28) | **0.003** |
| PUD |  |  |
| Non-hospitalized COVID | 1.01(0.81,1.25) | 0.935 |
| Hospitalized COVID | 1.13(0.60,2.14) | 0.701 |
| Severe COVID | 1.03(0.25,4.26) | 0.971 |
| GERD |  |  |
| Non-hospitalized COVID | 1.20(1.10,1.31) | **<0.001** |
| Hospitalized COVID | 1.78(1.38,2.29) | **<0.001** |
| Severe COVID | 2.14(1.01,4.53) | **0.047** |
| IBD |  |  |
| Non-hospitalized COVID | 1.08(0.77,1.53) | 0.644 |
| Hospitalized COVID | 1.78(0.78,4.10) | 0.172 |
| Severe COVID | NA | NA |
| Gallbladder disease |  |  |
| Non-hospitalized COVID | 1.03(0.89,1.19) | 0.654 |
| Hospitalized COVID | 1.96(1.40,2.75) | **<0.001** |
| Severe COVID | 2.66(0.90,7.84) | 0.076 |
| Severe liver disease |  |  |
| Non-hospitalized COVID | 0.92(0.66,1.28) | 0.622 |
| Hospitalized COVID | 2.80(1.65,4.73) | **<0.001** |
| Severe COVID | 3.41(0.41,28.40) | 0.257 |
| NAFLD |  |  |
| Non-hospitalized COVID | 1.61(1.35,1.91) | **<0.001** |
| Hospitalized COVID | 2.46(1.61,3.76) | **<0.001** |
| Severe COVID | 1.61(0.47,5.49) | 0.446 |
| Pancreatic disease |  |  |
| Non-hospitalized COVID | 1.16(0.91,1.48) | 0.232 |
| Hospitalized COVID | 3.37(2.28,4.99) | **<0.001** |
| Severe COVID | 3.30(0.54,20.10) | 0.195 |

GI, gastrointestinal;GERD, gastroesophageal reflux disease; PUD, peptic ulcer disease; IBD, inflammatory bowel disease; NAFLD, non-alcohol fatty liver disease;

HR: hazard ratio; CI: confidence interval;

Outcomes were ascertained 30 days after the COVID-19-positive test until the end of follow-up. Weighted HRs after IPTW and 95% CIs are presented.
